# Cardiovascular Toxicities of Ibrutinib: A Pharmacovigilance Study Based on the United States Food and Drug Administration Adverse Event Reporting System Database

**DOI:** 10.3390/ph16010098

**Published:** 2023-01-09

**Authors:** Yi Zheng, Xiaojing Guo, Chenxin Chen, Lijie Chi, Zhijian Guo, Jizhou Liang, Lianhui Wei, Xiao Chen, Xiaofei Ye, Jia He

**Affiliations:** Department of Health Statistics, Naval Medical University, 800 Xiangyin Road, Shanghai 200433, China

**Keywords:** ibrutinib, FAERS, cardiovascular events, disproportionality analysis, pharmacovigilance study

## Abstract

Background: Although ibrutinib has been widely used to treat haematological malignancies, many studies have reported associated cardiovascular events. These studies were primarily animal experiments and clinical trials. For more rational clinical drug use, a study based on post-marketing data is necessary. Aim: Based on post-marketing data, we investigated the clinical features, time to onset, and outcomes of potential cardiovascular toxicities of ibrutinib. Methods: This disproportionality study utilised data from the 2014–2021 United States Food and Drug Administration Adverse Event Reporting System (FAERS) database. We used two disproportionality methods information component (IC) and reporting odds ratio (ROR)) to detect the potential cardiovascular toxicities of ibrutinib. Positive signals were defined as IC_025_ > 0 and ROR_025_ > 1. Results: A total of 10 cardiovascular events showed positive signals: supraventricular tachyarrhythmias, haemorrhagic central nervous system vascular conditions, ventricular tachyarrhythmias, cardiac failure, ischaemic central nervous system vascular conditions, cardiomyopathy, conduction defects, myocardial infarction, myocardial infarction disorders of sinus node function, and torsade de pointes/QT prolongation. Cardiomyopathy and supraventricular tachyarrhythmias were the two most common signals. Disorders of sinus node function were observed for the first time, which may be a new adverse effect of ibrutinib. Conclusions: This pharmacovigilance study systematically explored the adverse cardiovascular events of ibrutinib and provided new safety signals based on past safety information. Attention should be paid to some high-risk signals.

## 1. Introduction

Ibrutinib, the first small molecule of Bruton’s tyrosine kinase inhibitor (BTKi), was approved by the United States Food and Drug Administration (FDA) in November 2013 for treating recurrent mantle cell lymphoma (MCL) [1,2]. As a BTKi, ibrutinib irreversibly inhibits BTK activity by highly specific and covalent binding to cysteine-481 (Cys-481) at the BTK active site, thereby blocking the activation of the B cell receptor signalling pathway. Consequently, it alters the tumour microenvironment, inhibits the malignant proliferation of tumour B cells, and induces apoptosis [3,4]. Ibrutinib is effective in various B-cell malignancies; its indications now include MCL, chronic lymphocytic leukaemia (CLL), small lymphocytic lymphoma with 17p deletion (SLL), marginal zone lymphoma, chronic graft versus host disease, and Waldenström’s macroglobulinemia (WM) [1,2,5,6,7]. The recommended dose for MCL treatment was 560 mg once daily, and 420 mg once daily was recommended for CLL and WM [8]. After being marketed, ibrutinib has recently become popular for its curative effect in treating haematological malignancies.

Despite its huge clinical benefits, ibrutinib inevitably causes drug toxicity in patients. Some recent studies have reported cardiovascular toxicities of ibrutinib, such as atrial fibrillation [9,10,11,12], supraventricular arrhythmias, and ventricular arrhythmias [13]. In severe cases, cardiovascular toxicity is life-threatening [14]. With the widespread clinical application of ibrutinib, it is necessary to explore the cardiovascular toxicities of ibrutinib based on post-marketing data and the time to onset (TTO) for the safe treatment of patients. This pharmacovigilance study based on the Adverse Event Reporting System Database of the US Food and Drug Administration (FAERS) was conducted to detect the potential cardiovascular toxicities of ibrutinib.

## 2. Results

### 2.1. Baseline Characteristics

After data cleaning, we extracted 78,887,460 records from the FAERS database from 1 January 2014 to 1 July 2021, of which 43,459 were submitted for ibrutinib, with 5974 records related to cardiovascular complications. Table 1 shows the baseline characteristics of patients treated with ibrutinib. The USA was the main reporter country (N = 31,962, 74.1%). Males (N = 25,179, 62.6%) and older adults aged >60 years (N = 20,410, 86%) were the main patients taking ibrutinib; they were also the main patients with cardiovascular complications. CLL was the top indication (N = 21,218, 48.9%), followed by MCL (N = 3728, 8.6%) and WM (N = 2636, 6.1%). The most common dosage for patients was 420 mg (N = 21,364, 62.3%).

### 2.2. Disproportionality Analysis

Overall, 10 cardiovascular-related standardised MedDRA Query (SMQ) showed signals: SVT, Haemorrhagic-CNS, VT, CF, Ischaemic-CN, CM, CD, MI, DSN, and Tdp/QTp. CM (N = 2641), SVT (N = 2557), and CF (N = 1887) were the three most common cardiovascular events (Table 2). We also explored the baseline characteristics of patients with the above cardiovascular events (Supplementary Table S1).

**Figure 1:**
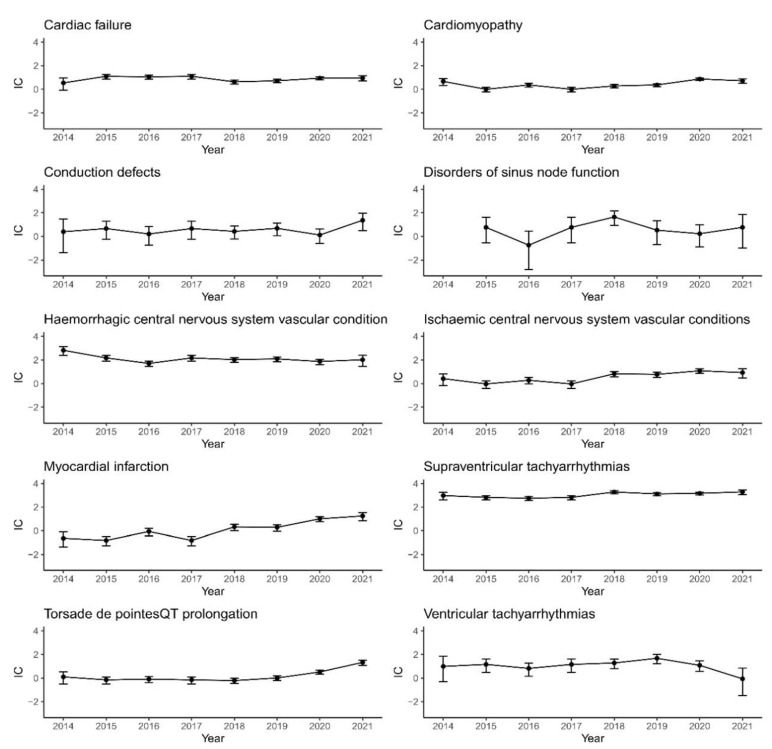
Information component (IC) and its 95% CI over time for 10 signals.

We further explored the changes in IC values and their 95% CIs from 2014 to 2021 for 10 cardiovascular events (Figure 1). We noticed that haemorrhagic-CNS and ST showed strong signals over the above years and did not change much; this should require additional attention.

**Figure 2:**
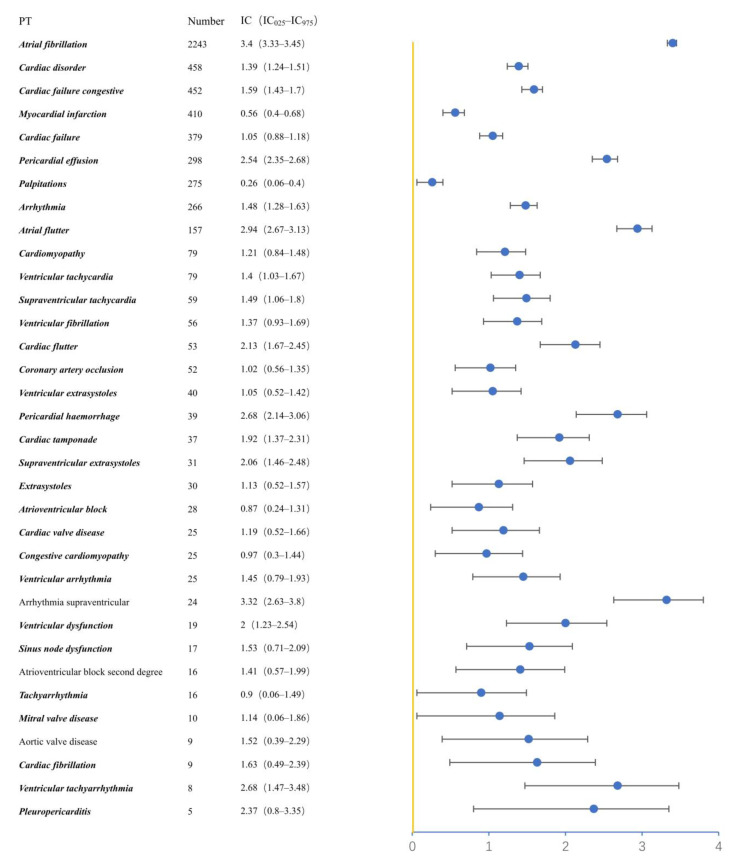
The signals in preferred terms (PTs) associated with cardiovascular toxicities.

We also explored the cardiovascular adverse events with the preferred term (PT). The signals can be accessed in Figure 2; 34 PTs were shown as signals. According to European Medicines Agency (EMA)’s Designated Medical Event list, 31 PTs were important medical events among the PTs above, except supraventricular arrhythmia, atrioventricular block (second degree), and aortic valve disease. Atrial fibrillation (AF) (N = 2243, IC_025_/ROR_025_ = 3.33/10.12) was the most common PT and the strongest signal. Cardiac disorder (N = 458, IC_025_/ROR_025_ = 1.24/2.40) was another common PT, followed by AF.

We explored the potential cardiac toxicity potential toxicity to other systems of ibrutinib (Supplementary Table S2).

### 2.3. TTO

**Figure 3:**
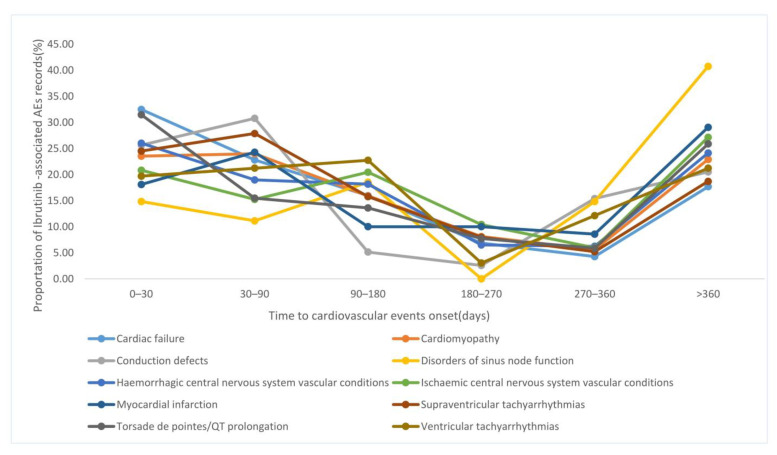
Time to onset for 10 cardiovascular toxicities.

Figure 3 presents the differential spectra of TTO of the 10 cardiovascular toxicities mentioned above. Overall, the median TTO of cardiovascular events was 99 days, and Q1–Q3 was 28–335 days. CD showed the shortest median time of 64 days (Q1–Q3: 26–322 days), followed by CF at 69 days (Q1–Q3: 19–224 days). DSN showed the longest median time of 282 days (Q1–Q3: 69–432 days).

### 2.4. Outcome

We further explored the outcomes of these 10 cardiovascular toxicities. Among the 10 cardiovascular toxicities, the proportion of death in CD (10.4%) was the lowest, and Tdp/QTP (35.3%) was the highest. Overall, the proportion of death in patient outcomes due to cardiovascular events was 21%.

## 3. Discussion

Ibrutinib is one of the most important therapies for patients with haematopoietic malignancies, particularly CLL [15]. In September 2022, the Pharmacovigilance Risk Assessment Committee published a ‘direct healthcare professional communications’ on the increased risk of fatal and serious cardiac arrhythmias and cardiac failure with the use of ibrutinib [16]. Therefore, it is important to detect potential cardiovascular toxicities to protect the patients better. To our knowledge, this study presents the most exhaustive and extensive characterisation of ibrutinib-associated cardiovascular toxicities based on the FAERS database. We detected 10 potential cardiovascular toxicities and some interesting findings as follows:

In this study, SVT was a common cardiovascular toxicity associated with ibrutinib; AF was the primary type. In a mice experiment, Xiao et al. suggested that ibrutinib inhibits C-terminal Src kinase (CSK) to cause AF [17]. CSK decreases Src family tyrosine kinase (SFK) activity via C-terminal phosphorylation. SFKs are involved in many cellular functions, including differentiation, cell proliferation, migration, survival, adhesion, inflammation, and programmed cell death [18]. The balance between SFK activation and CSK phosphorylation can be maintained. Conversely, it can be harmful to health. Compared with the ventricle, CSK was more enriched in the atria. Pharmacokinetic studies have shown that ibrutinib causes CSK to exceed its IC_50_ continuously [19]. The study suggested that it may be because ibrutinib increases SFK activity via CSK, leading to increased inflammation and fibrosis, predisposing the heart to AF [17]. Another study suggested that ibrutinib causes AF via the phosphoinositide 3-kinase (PI3K)-Akt pathway [20].

AF can trigger other cardiovascular events, such as ischaemic CNS. AF results in altered haemodynamics within the heart, producing emboli that block arteries of various sizes with blood flow [21]. We noticed that the median TTO of Ischaemic-CNS (269 days) was longer than that of SVT (81 days), further confirming the possibility of this mechanism. The mechanism of AF is unclear and requires further research.

Tdp/QTp is a special type of VT with a higher death proportion (N = 372, 35.3%). Male sex, ischaemic heart disease, prolonged QT interval, previous AF, diabetes, and valvular disease were associated with an increased risk of VT [22]. According to a previous study, ibrutinib may influence adenosine monophosphate-activated protein kinase to mediate the dysregulation of calcium-handling proteins to cause VT, as it activates Akt under metabolic stress [23]. Another study on rats showed that acute treatment with ibrutinib may enhance spatially discordant action potential duration alternans, a recognised risk factor for arrhythmias [24]. Ventricular fibrillation (VF) (N = 56, IC_025_ = 0.93) was also a signal of PT. It is one of the most serious life-threatening heart diseases; VF can lead to sudden death and high mortality [25,26]. Although the mechanism is unclear, the high death rate should be considered, particularly in older male patients, and early treatment should be provided.

Our study identified DSN as a newer cardiovascular toxicity of ibrutinib. Several cases have been reported [27]. Sinus node dysfunction (N = 17, IC_025_/ROR_025_ = 0.71/1.79) as a PT was also a signal, demonstrating the adverse effect of ibrutinib on the sinus node. We also found that males (80.0%) were the majority, significantly higher than 60.6% (the value of all cardiovascular events). The median TTO for DSN was the longest among the 10 cardiovascular events.

CM was another common cardiovascular toxicity in our study. Several relevant cases have been reported [28,29]. The mechanism may be as follows: ibrutinib inhibits protein kinase C (PKC) via inhibition of BTK, and this may lead to increased L-type calcium activity, which is implicated in increased myocardial contractility [29,30]. CM can also be caused by AF, tachycardia, and hypertension [15]. CM is generally accompanied by structural changes in the heart, which require attention.

HF is a life-threatening disease that requires early management. We found that the TTO of HF was 69 days, which was shorter than for all cardiovascular events (99 days). HF can be caused by other cardiovascular events such as HF, VT, and CM. FDA label showed that HF occurred particularly in patients with acute infections, cardiac risk factors, a previous history of cardiac arrhythmias, and hypertension. A previous study suggested that using amiodarone (one of the most common antiarrhythmic drugs) could maintain the serum level of ibrutinib, thus increasing its toxicity [31]. HF relapse rapidly leads to physical health deterioration; therefore, early treatment and management are necessary.

Haemorrhage is another common side effect of ibrutinib [32]. In our study, we found that haemorrhage (N = 1093, IC_025_ = 2.36) is a signal of PT with high frequency. We further analysed the platelet count; it decreased (N = 1127, IC_025_ = 1.90) as PT was also a positive signal with high frequency. As a BTKi, in addition to inhibiting BTK, ibrutinib can inhibit several other intracellular molecules important for platelet signalling, including tyrosine kinase expressed in hepatocellular carcinoma (Tec) [33]. BTK and Tec can lead to a decrease in platelet count via C-type lectin-like receptor 2 (CLEC-2) and the platelet collagen receptor glycoprotein VI (GPVI) pathways [32]. Another study showed that ibrutinib inhibits platelet adhesion to fibrinogen by inhibiting the αIIbβ3 outside-in signalling pathway [34]. The decreased platelet count can cause bleeding, which may be a mechanism leading to haemorrhage-CNS. Haemorrhage-CNS is a special type that can cause sudden death and other serious complications; this needs attention.

Hypertension (HTN) (N = 1299, IC_025_/ROR_025_ = −0.14/0.92) was a signal as SMQ. However, it showed a positive PT signal (N = 661, IC_025_/ROR_025_ = 0.12/1.10). An SMQ contains several PTs, which describe the approximate disease condition. The above situations proved that HTN was not a robust signal. Several studies suggested that HTN is an adverse effect of ibrutinib [35,36]. The ibrutinib FDA label also indicated that in clinical trials of 1476 patients who received ibrutinib, HTN could occur in 19% of patients, and 8% of patients could experience grade 3 or higher HTN. The exact relationship between ibrutinib and HTN and the relevant mechanisms require further prospective studies.

Compared with a 2019 study based on the Vigibase database exploring the cardiotoxicities of the ibrutinib [13], we additionally found MI, CM, Tdp/QTp, and DSN as four potential cardiotoxicities. This may be due to the difference between the two databases and the larger amount of data in this study through the second quarter of 2021. According to another 2022 study, the second generation BTKi was safer than ibrutinib, which was the first generation BTKi, especially in cardiotoxicity [37].

## 4. Limitations

This study has several limitations that should be acknowledged. First, the FAERS database is based on spontaneous data. Thus, intrinsic bias, such as under-reporting, over-reporting, and incomplete information, is inevitable and unquantifiable [38]. Second, FAERS cannot evaluate the exact number of patients treated with ibrutinib; therefore, we could not determine the incidence of cardiovascular events [39]. Further, the reporting behaviour could be affected by changing the awareness of toxicities over time. Moreover, some very close adverse events were identified by different PTs, which were considered distinct adverse events, possibly impacting the accuracy of the results. Moreover, although disproportionality methods are efficient, we should also recognise the shortcomings in dealing with confounding factors, such as co-prescription and masking effects [40], and we did not consider a stratified analysis. Disproportionality methods indicate potential safety issues, which should be validated and followed up in prospective studies. 

## 5. Methods

### 5.1. Study Design and Database

This observational pharmacovigilance study utilised the FAERS database and covered the period from 1 January 2014 to 30 June 2021. The FAERS database is an open database maintained by the FDA; it collects adverse event (AE) reports from different sources, including patients, healthcare professionals, and drug manufacturers [38]. It is one of the most common databases for mining adverse drug reaction signals in pharmacovigilance.

### 5.2. Data Cleaning

Before data analysis, reports with the same sex, age, reporting country, adverse events, drug name, starting time, and ending time were defined as repeated data and underwent deduplication processing. No imputation method for missing data was used in this study because FAERS is a spontaneous database with a large proportion of missing data for the variables. Both generic and brand names were used to identify the target drug, ibrutinib. AEs were coded with the PTs in FAERS, according to the Medical Dictionary for Regulatory Activities (MedDRA). Several PTs can be grouped into a SMQ and a System Organ Class (SOC) to describe a disease condition or a systemic disease [41]. In this study, we used 16 SMQs related to cardiovascular events (Table 2) as AEs and the PTs related to the SOC of Cardiac disorders (Code: 10007541) to detect the cardiovascular toxicities associated with ibrutinib. TTO was defined as the time from the start date of ibrutinib treatment to the onset date of cardiovascular events. We used the median days and its quarter 1–3 (Q1–Q3) to demonstrate the TTO of the cardiac toxicities.

### 5.3. Statistical Analysis

Disproportionality analysis, also called case/non-case analysis, was used in this study, which is also the most common signal detection method in pharmacovigilance [42]. The reporting odds ratio (ROR) and the information component (IC) are two frequently applied disproportionality analysis methods [43,44]. Both methods were used to detect signals in this study. Using statistical shrinkage transformation can reduce false-negative signals and obtain robust results [45]. In this study, the shrunken IC and ROR were calculated as follows:

$$\mathrm{IC}=\log_{2}\frac{N_{\mathrm{observed}}+0.5}{N_{\mathrm{expected}}+0.5}$$

$$\mathrm{ROR}=\frac{N_{\mathrm{observed}}+0.5}{N_{\mathrm{expected}}+0.5}$$

$$N_{\mathrm{expected}}=\frac{N_{\mathrm{drug}}\times N_{\mathrm{event}}}{N_{\mathrm{total}}}$$

where N_observed_ is the observed number of records of the target drug AEs, N_expected_ is the expected number of records of the target drug-AE combination, N_drug_ is the total number of records of the target drug, N_event_ is the total number of records of target AEs, and N_total_ is the total number of records in the entire database.

A signal was shown when the lower limit of the 95% confidence interval of IC (IC_025_) exceeded 0 or the lower limit of the 95% confidence interval of ROR (ROR_025_) exceeded 1, with N_expected_ > 3. All analyses were performed using SAS version 9.4 (SAS Institute, Inc., Cary, NC, USA).

## 6. Conclusions

Comprehensive pharmacovigilance analysis contributes to a further understanding of the cardiovascular safety of ibrutinib. CF, CM, CD, DSN, MI, SVT, Tdp/QTp, VT, ischaemic CNS, and haemorrhagic-CNS were 10 potential cardiovascular events related to ibrutinib in our study. DSN was first identified as a potential cardiovascular toxicity of ibrutinib in our study; this requires further investigation. If identified, the Marketing Authorization Holder (MAH)/Regulatory also needs to be warranted. For medication safety, the cardiovascular events mentioned above require constant attention in patients taking ibrutinib.

## Figures and Tables

**Table 1:** Characteristics of records associated with Ibrutinib in FAERS from 2014–2021.

Characteristics	Ibrutinib
Sex	
Data available	40,230
Male	25,179 (62.6)
Female	15,051 (37.4)
Age	
Data available	23,721
<45	487 (2.1)
<60	2824 (11.9)
≥60	20,410 (86.0)
Report country	
Data available	43,149
USA	31,962 (74.1)
France	1540 (3.6)
Canada	1388 (3.2)
UK	1012 (2.3)
Germany	910 (2.1)
Others	6337 (14.7)
Outcome	
Data available	34,235
Hospitalisation—Initial or Prolonged	14,167 (41.4)
Death	7178 (21.0)
Disability	272 (0.8)
Life-Threatening	253 (0.7)
Other Serious (Important Medical Event)	12,365 (36.1)
Indication	
Data available	43,350
CLL	21,218 (48.9)
MCL	3728 (8.6)
WM	2636 (6.1)
NHL	864 (2.0)
B-CLL	790 (1.8)
Others	14,114 (32.6)
Doses	
Data available	34,281
140	3690 (10.8)
280	4019 (11.7)
420	21,364 (62.3)
560	4384 (12.8)
Others	824 (2.4)
TTO	
Data available	2592
Median days	99
Q1–Q3 *	28–335

*: Q1–Q3: Quarter 1–3.

**Table 2:** Cardiovascular events in standardised MedDRA Query (SMQ) with disproportionality analysis in United States Food and Drug Administration Adverse Event Reporting System (FAERS) database.

SMQ	Frequency	IC (95%CI)	ROR (95%CI)	SMQ Code
Supraventricular tachyarrhythmias	2557	3.09 (3.02–3.13)	8.49 (8.16–8.83)	20000057
Haemorrhagic central nervous system vascular conditions	1080	2.13 (2.03–2.2)	4.37 (4.11–4.64)	20000064
Ventricular tachyarrhythmias	226	1.18 (0.96–1.34)	2.27 (1.99–2.58)	20000058
Cardiac failure	1887	0.92 (0.84–0.97)	1.89 (1.8–1.98)	20000004
Ischaemic central nervous system vascular conditions	899	0.74 (0.63–0.82)	1.67 (1.57–1.79)	20000063
Disorders of sinus node function	51	0.53 (0.07–0.87)	1.45 (1.1–1.91)	20000055
Cardiomyopathy	2641	0.49 (0.43–0.54)	1.41 (1.35–1.46)	20000150
Conduction defects	130	0.49 (0.2–0.7)	1.4 (1.18–1.66)	20000056
Myocardial infarction	629	0.26 (0.12–0.35)	1.19 (1.1–1.29)	20000047
Torsade de pointes/QT prolongation	1104	0.17 (0.07–0.24)	1.12 (1.06–1.19)	20000001
Embolic and thrombotic events, arterial	136	0 (−0.28–0.21)	1 (0.85–1.19)	20000082
Embolic and thrombotic events, venous	433	0 (−0.16–0.12)	1 (0.91–1.1)	20000083
Hypertension	1299	−0.05 (−0.14–0.02)	0.97 (0.92–1.02)	20000147
Conditions associated with central nervous system haemorrhages and cerebrovascular accidents	200	−0.17 (−0.41–0)	0.89 (0.77–1.02)	20000166
Pulmonary hypertension	1436	−0.38 (−0.47–−0.32)	0.77 (0.73–0.81)	20000130
Embolic and thrombotic events, vessel type unspecified and mixed arterial and venous	344	−0.67 (−0.85–−0.54)	0.63 (0.56–0.7)	20000083

## Data Availability

The FAERS database utilised in this study is available at: https://fis.fda.gov/extensions/FPD-QDE-FAERS/FPD-QDE-FAERS.html, accessed on 8 January 2023).

## Supplementary Materials

https://www.mdpi.com/article/10.3390/ph16010098/s1, Table S1: Characteristics of patients with cardiovascular events in FAERS from 2014–2021; Table S2: Details of all signals in PTs.

## Abbreviation

Adverse event	Abbreviation
Supraventricular tachyarrhythmias	SVT
Haemorrhagic central nervous system vascular conditions	Haemorrhagic-CNS
Ventricular tachyarrhythmias	VT
Cardiac failure	CF
Ischaemic central nervous system vascular conditions	Ischaemic-CNS
Disorders of sinus node function	DSN
Cardiomyopathy	CM
Conduction defects	CD
Myocardial infarction	MI
Torsade de pointes/QT prolongation	Tdp/QTp
