# Supplementary material for: Cardiovascular Toxicities of Ibrutinib: A Pharmacovigilance Study Based on the United States Food and Drug Administration Adverse Event Reporting System Database
Source: Pharmaceuticals (Basel). 2023 Jan 9;16(1):98. doi: 10.3390/ph16010098 (PMC9863914; doi:10.3390/ph16010098)
Supplement: Supplementary file 1 [file pharmaceuticals-16-00098-s001.zip › pharmaceuticals-1970457-supplementary .pdf]

Table S1. Characteristics of patients with cardiovascular events in FAERS from 2014–2021.

Characteristics	CF	CM	CD	DSN	MI	SVT	Tdp/QTp	VT	Ischaemic-CNS	Haemorrhagic-CNS
Sex										
Data available	1751	2213	100	45	564	2115	988	177	792	883
Male	1017(58.1)	1447(65.4)	74(74.0)	36(80.0)	395(70.0)	1450(68.6)	656(66.4)	140(79.1)	498(62.9)	592(67.0)
Femal	734(41.9)	766(34.6)	37(37.0)	9(20.0)	169(30.0)	665(31.4)	332(33.6)	37(20.9)	294(37.1)	291(33.0)
Age										
Data available	1215	1622	88	42	404	1620	747	144	574	708
<45	5(0.4)	21(1.3)	1(1.1)	0(0.0)	1(0.2)	6(0.4)	11(1.5)	1(0.7)	5(0.9)	9(1.3)
<60	99(8.1)	183(11.3)	1(1.1)	9(21.4)	37(9.2)	109(6.7)	109(14.6)	31(21.5)	34(5.9)	59(8.3)
⩾60	1111(91.4)	1418(87.4)	86(97.7)	33(78.6)	366(90.6)	1505(92.9)	627(83.9)	112(77.8)	535(93.2)	640(90.4)
Data available	1795	2403	117	49	600	2442	1061	206	849	1009
USA	1482(82.6)	1674(69.7)	71(60.7)	30(61.2)	409(68.2)	1706(69.9)	693(65.3)	127(61.7)	608(71.6)	551(54.6)
France	39(2.2)	127(5.3)	11(9.4)	1(2.0)	11(1.8)	139(5.7)	39(3.7)	9(4.4)	32(3.8)	67(6.6)
Canada	84(4.7)	102(4.2)	1(0.9)	0(0.0)	21(3.5)	56(2.3)	54(5.1)	7(3.4)	21(2.5)	34(3.4)
UK	18(1.0)	53(2.2)	4(3.4)	1(2.0)	19(3.2)	72(2.9)	41(3.9)	10(4.9)	26(3.1)	68(6.7)
Germany	17(0.9)	67(2.8)	4(3.4)	2(4.1)	18(3.0)	62(2.5)	20(1.9)	6(2.9)	21(2.5)	38(3.8)
Others	155(8.6)	380(15.8)	26(22.2)	15(30.6)	122(20.3)	407(16.7)	214(20.2)	47(22.8)	141(16.6)	251(24.9)
Outcome										
Data available	1420	2247	115	47	602	2203	1054	203	855	1017

Hospitalization - Initial or Prolonged	739(52.0)	1177(52.4)	58(50.4)	26(55.3)	298(49.5)	1186(53.8)	449(42.6)	94(46.3)	415(48.5)	448(44.1)
Death	164(d11.5)	352(15.7)	12(10.4)	9(19.1)	159(26.4)	291(13.2)	372(35.3)	40(19.7)	166(19.4)	274(26.9)
Disability	11(0.8)	16(0.7)	0(0.0)	0(0.0)	4(0.7)	20(0.9)	3(0.3)	2(1.0)	12(1.4)	41(4.0)
Life-Threatening	7(0.5)	31(1.4)	5(4.3)	0(0.0)	5(0.8)	25(1.1)	8(0.8)	6(3.0)	5(0.6)	20(2.0)
Other Serious (Important Medical Event)	499(35.1)	671(29.9)	40(34.8)	12(25.5)	136(22.6)	681(30.9)	222(21.1)	61(30.0)	257(30.1)	234(23.0)
Indication										
Data available	1794	2406	116	49	603	2446	1063	204	856	1017
CLL	1025(57.1)	1311(54.5)	69(59.5)	32(65.3)	363(60.2)	1300(53.1)	589(55.4)	118(57.8)	510(59.6)	526(51.7)
MCL	151(8.4)	257(10.7)	6(5.2)	6(12.2)	61(10.1)	248(10.1)	95(8.9)	18(8.8)	69(8.1)	114(11.2)
WM	137(7.6)	137(5.7)	13(11.2)	1(2.0)	28(4.6)	171(7.0)	63(5.9)	16(7.8)	51(6.0)	54(5.3)
NHL	33(1.8)	46(1.9)	0(0.0)	0(0.0)	7(1.2)	37(1.5)	25(2.4)	2(1.0)	17(2.0)	18(1.8)
B-CLL	37(2.1)	48(2.0)	1(0.9)	0(0.0)	10(1.7)	41(1.7)	13(1.2)	2(1.0)	15(1.8)	12(1.2)
Others	411(22.9)	607(25.2)	27(23.3)	10(20.4)	134(22.2)	649(26.5)	278(26.2)	48(23.5)	194(22.7)	291(28.6)
Doses										
Data available	3781	3948	179	73	833	3663	1504		1136	1108
140	603(15.9)	558(14.1)	31(17.3)	15(20.5)	75(9.0)	487(13.3)	141(9.4)	35(13.5)	136(12.0)	104(9.4)
280	716(18.9)	693(17.6)	37(20.7)	6(8.2)	115(13.8)	672(18.3)	237(15.8)	36(13.8)	167(14.7)	139(12.5)
420	2092(55.3)	2149(54.4)	95(53.1)	43(58.9)	539(64.7)	2024(55.3)	906(60.2)	143(55.0)	716(63.0)	657(59.3)

560	311(8.2)	458(11.6)	12(6.7)	6(8.2)	89(10.7)	385(10.5)	179(11.9)	34(13.1)	98(8.6)	161(14.5)
Others	59(1.6)	90(2.3)	4(2.2)	3(4.1)	15(1.8)	95(2.6)	41(2.7)	12(4.6)	18(1.6)	47(4.2)
TTO										
Data available	351	642	39	27	210	653	375	66	269	369
Median days	69	106	64	282	143	81	114	131	141	111
Q1-Q3	19-224	33-317	26-322	69-432	40-432	30-248	19-368	37-313	39-406	28-340

Table S2 Details of all signals in PTs

PT	Frequency	IC	IC_{025}	IC_{975}	ROR	ROR_{025}	ROR_{975}
Death	3909	1.18	1.12	1.22	2.26	2.19	2.33
Fatigue	2913	0.64	0.58	0.68	1.55	1.50	1.61
Diarrhoea	2635	0.82	0.76	0.87	1.77	1.70	1.84
Incorrect dose administered	2605	2.98	2.92	3.03	7.92	7.61	8.23
Atrial fibrillation	2243	3.40	3.33	3.45	10.55	10.12	11.01
Pneumonia	2054	1.32	1.25	1.38	2.50	2.39	2.61
Contusion	1865	2.99	2.92	3.05	7.97	7.61	8.34
Asthenia	1507	0.85	0.77	0.92	1.81	1.72	1.90
Fall	1430	0.89	0.81	0.96	1.86	1.76	1.96
Arthralgia	1341	0.42	0.33	0.49	1.34	1.27	1.41
Rash	1338	0.42	0.33	0.48	1.34	1.27	1.41
Pyrexia	1224	0.51	0.42	0.58	1.42	1.35	1.51
Disease progression	1132	2.28	2.18	2.35	4.85	4.57	5.14
Platelet count decreased	1127	2.00	1.90	2.07	4.01	3.78	4.25
Muscle spasms	1117	1.51	1.41	1.58	2.85	2.69	3.03
Haemorrhage	1093	2.46	2.36	2.53	5.50	5.18	5.84
Dizziness	1089	0.10	0.00	0.18	1.07	1.01	1.14
Peripheral swelling	1000	0.98	0.88	1.06	1.98	1.86	2.10
White blood cell count increased	931	3.25	3.14	3.32	9.48	8.89	10.12
Haemoglobin decreased	911	1.77	1.66	1.84	3.40	3.18	3.63
Pleural effusion	902	2.57	2.46	2.65	5.95	5.58	6.36
Weight decreased	830	0.23	0.12	0.31	1.17	1.10	1.26
Anaemia	811	0.59	0.47	0.67	1.50	1.40	1.61
Hospitalisation	809	1.72	1.61	1.81	3.31	3.08	3.54
Urinary tract infection	781	1.03	0.91	1.12	2.04	1.90	2.19
Product use in unapproved indication	771	0.23	0.11	0.32	1.17	1.09	1.26
Decreased appetite	761	0.42	0.30	0.51	1.34	1.25	1.44

White blood cell count decreased	755	1.46	1.34	1.55	2.76	2.56	2.96
Cough	755	0.15	0.03	0.23	1.11	1.03	1.19
Epistaxis	742	1.90	1.78	1.99	3.73	3.47	4.01
Myalgia	713	1.07	0.95	1.16	2.10	1.95	2.26
Constipation	672	0.34	0.21	0.43	1.27	1.17	1.37
Product use issue	671	0.14	0.02	0.24	1.11	1.02	1.19
Hypertension	661	0.24	0.12	0.34	1.18	1.10	1.28
Sepsis	622	1.02	0.89	1.12	2.03	1.87	2.19
Thrombocytopenia	622	0.96	0.82	1.05	1.94	1.79	2.10
Infection	615	0.62	0.48	0.71	1.53	1.42	1.66
Surgery	610	2.84	2.71	2.94	7.17	6.62	7.77
Stomatitis	605	1.68	1.54	1.77	3.20	2.95	3.46
Neutropenia	601	0.64	0.50	0.74	1.56	1.44	1.69
Lymphadenopathy	519	2.58	2.44	2.69	5.98	5.48	6.52
Cerebrovascular accident	505	0.92	0.78	1.03	1.90	1.74	2.07
Oedema peripheral	489	0.92	0.77	1.03	1.89	1.73	2.07
Dehydration	485	0.72	0.57	0.83	1.65	1.51	1.80
Cataract	476	1.76	1.61	1.87	3.39	3.09	3.71
Cardiac disorder	458	1.39	1.24	1.51	2.63	2.40	2.88
Cardiac failure congestive	452	1.59	1.43	1.70	3.00	2.74	3.29
Increased tendency to bruise	416	4.20	4.04	4.32	18.39	16.67	20.28
Haematuria	412	2.09	1.93	2.21	4.26	3.86	4.69
Myocardial infarction	410	0.56	0.40	0.68	1.47	1.34	1.62
Dyspepsia	396	0.84	0.67	0.96	1.79	1.62	1.97
Blood count abnormal	394	1.89	1.73	2.01	3.71	3.36	4.10
Adverse drug reaction	383	0.98	0.81	1.10	1.97	1.78	2.18
Cardiac failure	379	1.05	0.88	1.18	2.07	1.88	2.30
Petechiae	378	3.88	3.71	4.00	14.73	13.30	16.32
Adverse event	378	0.98	0.81	1.11	1.98	1.79	2.19
Chronic lymphocytic leukaemia	363	4.85	4.68	4.97	28.82	25.92	32.05
Onychoclasis	351	4.21	4.04	4.34	18.54	16.66	20.63
Febrile neutropenia	351	0.54	0.36	0.67	1.45	1.31	1.62
Haematoma	346	2.30	2.13	2.43	4.94	4.44	5.49
Loss of consciousness	346	0.38	0.20	0.51	1.30	1.17	1.45
Gastrointestinal haemorrhage	343	0.36	0.18	0.49	1.28	1.15	1.43
Cerebral haemorrhage	341	2.14	1.96	2.27	4.41	3.96	4.91
Bone pain	327	1.35	1.16	1.48	2.54	2.28	2.83
Sinusitis	323	0.22	0.03	0.35	1.16	1.04	1.30
Muscular weakness	315	0.31	0.13	0.45	1.24	1.11	1.39
Neuropathy peripheral	315	0.25	0.06	0.39	1.19	1.06	1.33
Pulmonary oedema	313	1.62	1.43	1.75	3.07	2.74	3.43
Syncope	312	0.56	0.38	0.70	1.48	1.32	1.65

Subdural haematoma	308	3.14	2.95	3.28	8.83	7.89	9.88
Respiratory failure	305	0.78	0.59	0.92	1.72	1.54	1.92
Red blood cell count decreased	300	1.89	1.70	2.02	3.70	3.30	4.14
Chills	299	0.32	0.13	0.46	1.25	1.12	1.40
Pericardial effusion	298	2.54	2.35	2.68	5.83	5.20	6.54
Cellulitis	292	1.24	1.04	1.38	2.36	2.10	2.64
Dry mouth	288	0.77	0.58	0.91	1.70	1.52	1.91
Dysphagia	282	0.52	0.32	0.66	1.43	1.27	1.61
Palpitations	275	0.26	0.06	0.40	1.20	1.06	1.35
Herpes zoster	272	0.72	0.51	0.86	1.64	1.46	1.85
Neutrophil count decreased	269	1.11	0.90	1.25	2.15	1.91	2.43
Vision blurred	269	0.19	-0.02	0.33	1.14	1.01	1.28
Arrhythmia	266	1.48	1.28	1.63	2.79	2.47	3.14
Product dose omission	263	0.27	0.06	0.42	1.20	1.07	1.36
Balance disorder	260	0.31	0.10	0.45	1.24	1.09	1.40
Thrombosis	259	0.58	0.38	0.73	1.50	1.33	1.69
Bronchitis	256	0.21	0.00	0.35	1.15	1.02	1.30
Hip fracture	255	2.06	1.85	2.21	4.17	3.68	4.71
White blood cell count abnormal	254	3.64	3.43	3.79	12.45	10.99	14.10
Haemoptysis	253	1.74	1.53	1.89	3.34	2.95	3.78
Rash macular	246	1.92	1.71	2.07	3.78	3.34	4.29
Fluid retention	238	0.76	0.55	0.92	1.69	1.49	1.92
Eye haemorrhage	229	3.03	2.81	3.18	8.15	7.15	9.29
Laboratory test abnormal	222	1.75	1.53	1.91	3.36	2.95	3.84
Rash pruritic	220	0.84	0.62	1.00	1.79	1.57	2.04
Chronic obstructive pulmonary disease	217	0.78	0.56	0.94	1.72	1.50	1.96
Tumour lysis syndrome	216	3.03	2.80	3.19	8.16	7.13	9.33
Skin discolouration	214	1.13	0.91	1.30	2.20	1.92	2.51
Skin lesion	212	1.46	1.23	1.63	2.75	2.41	3.15
Skin cancer	204	1.84	1.60	2.00	3.57	3.11	4.10
Pancytopenia	203	0.41	0.17	0.57	1.33	1.15	1.52
Haematochezia	202	0.79	0.56	0.96	1.73	1.51	1.99
Impaired healing	201	1.19	0.96	1.36	2.28	1.99	2.62
Post procedural haemorrhage	199	2.95	2.72	3.12	7.73	6.72	8.89
Upper respiratory tract infection	198	0.52	0.29	0.69	1.44	1.25	1.65
Basal cell carcinoma	196	1.56	1.33	1.73	2.95	2.57	3.40
Blister	193	0.40	0.16	0.58	1.32	1.15	1.52
Skin ulcer	192	1.08	0.84	1.26	2.12	1.84	2.44
Night sweats	190	1.32	1.08	1.49	2.50	2.16	2.88
Lung disorder	189	0.56	0.32	0.74	1.48	1.28	1.71

Lymphocytosis	188	4.96	4.72	5.14	31.20	26.91	36.17
Transient ischaemic attack	187	1.63	1.39	1.80	3.09	2.67	3.57
COVID-19	186	0.80	0.55	0.97	1.74	1.50	2.01
Septic shock	186	0.44	0.20	0.61	1.35	1.17	1.56
Lymphocyte count increased	180	4.21	3.97	4.39	18.53	15.97	21.51
Splenomegaly	178	2.34	2.09	2.52	5.05	4.36	5.86
Nephrolithiasis	176	0.71	0.46	0.89	1.63	1.41	1.89
Pneumonitis	175	1.45	1.20	1.63	2.73	2.35	3.16
Renal disorder	175	0.74	0.49	0.92	1.67	1.44	1.94
Localised infection	172	1.55	1.30	1.73	2.93	2.52	3.40
Gout	170	2.02	1.77	2.20	4.06	3.49	4.72
Cystitis	168	0.94	0.69	1.13	1.92	1.65	2.24
Leukocytosis	166	1.99	1.74	2.18	3.99	3.42	4.64
Blood urine present	163	1.92	1.66	2.11	3.78	3.24	4.41
Flatulence	160	0.52	0.26	0.71	1.43	1.23	1.67
Skin haemorrhage	159	2.56	2.30	2.75	5.91	5.06	6.92
Rectal haemorrhage	159	0.50	0.24	0.69	1.42	1.21	1.66
Atrial flutter	157	2.94	2.67	3.13	7.67	6.55	8.98
Head injury	156	1.21	0.94	1.40	2.31	1.97	2.71
Haemorrhage intracranial	155	2.17	1.91	2.36	4.51	3.85	5.28
Haemorrhagic diathesis	154	3.85	3.58	4.04	14.39	12.26	16.89
Nail disorder	152	2.60	2.33	2.79	6.05	5.15	7.10
Dry eye	152	0.84	0.57	1.03	1.79	1.52	2.10
Blood blister	149	4.33	4.06	4.53	20.18	17.12	23.78
Hospice care	146	3.54	3.27	3.74	11.65	9.88	13.73
Heart rate irregular	146	1.32	1.05	1.52	2.50	2.12	2.94
Rash erythematous	142	0.57	0.29	0.77	1.48	1.26	1.75
Ecchymosis	141	3.11	2.83	3.31	8.63	7.30	10.19
Dysuria	140	0.74	0.46	0.94	1.67	1.41	1.97
Immunodeficiency	139	1.38	1.10	1.59	2.61	2.21	3.08
Mantle cell lymphoma	138	5.27	4.99	5.48	38.68	32.48	46.05
Staphylococcal infection	136	0.82	0.53	1.02	1.76	1.49	2.09
Blood sodium decreased	135	1.93	1.65	2.14	3.81	3.22	4.51
Pulmonary mass	135	1.33	1.04	1.53	2.51	2.12	2.97
Blood potassium decreased	134	1.03	0.74	1.23	2.04	1.72	2.42
Platelet count abnormal	130	3.21	2.92	3.42	9.27	7.79	11.03
Hernia	128	1.55	1.26	1.77	2.94	2.47	3.50
Lung infection	126	1.01	0.71	1.22	2.01	1.69	2.39
Bronchopulmonary aspergillosis	125	1.90	1.60	2.11	3.72	3.12	4.44
Mass	125	1.64	1.35	1.86	3.12	2.62	3.72
Skin disorder	125	0.82	0.52	1.03	1.77	1.48	2.11
Pollakiuria	124	0.53	0.23	0.74	1.44	1.21	1.72
Fungal infection	122	0.61	0.31	0.83	1.53	1.28	1.83

Haemothorax	121	4.08	3.78	4.30	16.90	14.10	20.26
Viral infection	121	0.54	0.24	0.75	1.45	1.21	1.73
Aspergillus infection	118	1.91	1.61	2.13	3.77	3.14	4.52
Urinary retention	118	0.56	0.26	0.78	1.48	1.23	1.77
Skin fissures	116	1.82	1.51	2.04	3.53	2.94	4.23
Colitis	116	0.38	0.07	0.60	1.30	1.08	1.56
Gingival bleeding	115	1.92	1.61	2.14	3.77	3.14	4.53
Spinal fracture	115	1.50	1.19	1.72	2.83	2.36	3.40
Respiratory disorder	114	0.38	0.07	0.61	1.30	1.08	1.57
Lymphocyte count decreased	113	0.95	0.63	1.17	1.93	1.60	2.32
Clostridium difficile infection	113	0.63	0.32	0.86	1.55	1.29	1.87
Richter's syndrome	111	4.92	4.61	5.15	30.31	25.00	36.75
Squamous cell carcinoma	110	2.16	1.84	2.38	4.46	3.70	5.38
Transfusion	110	2.05	1.74	2.28	4.15	3.44	5.01
Deafness	110	0.96	0.64	1.18	1.94	1.61	2.34
Ear infection	106	0.75	0.43	0.98	1.68	1.39	2.04
Illness	106	0.31	-0.01	0.54	1.24	1.02	1.50
Ear haemorrhage	105	4.27	3.95	4.51	19.33	15.90	23.50
Rash papular	105	1.14	0.81	1.37	2.20	1.81	2.66
Haemoglobin abnormal	104	2.72	2.40	2.95	6.59	5.43	8.00
Blood iron decreased	104	1.69	1.36	1.92	3.22	2.65	3.90
Hypothyroidism	103	0.34	0.02	0.58	1.27	1.05	1.54
Acute respiratory failure	100	1.12	0.79	1.36	2.18	1.79	2.65
Respiratory tract infection	100	0.43	0.10	0.67	1.35	1.11	1.64
Autoimmune haemolytic anaemia	99	3.12	2.79	3.36	8.69	7.12	10.60
Blood pressure abnormal	98	1.11	0.78	1.35	2.16	1.77	2.63
Oral pain	98	0.87	0.53	1.11	1.82	1.50	2.22
Hypogammaglobulinaemia	97	2.42	2.08	2.66	5.35	4.38	6.53
Purpura	97	2.12	1.79	2.36	4.35	3.56	5.32
Nodule	97	1.16	0.82	1.40	2.23	1.83	2.72
Knee arthroplasty	96	0.35	0.02	0.60	1.28	1.05	1.56
Sinus disorder	95	1.01	0.67	1.25	2.01	1.64	2.46
Rash generalised	95	0.92	0.58	1.16	1.89	1.54	2.31
Haemorrhage subcutaneous	94	3.47	3.12	3.71	11.05	9.00	13.55
Faeces discoloured	94	1.11	0.77	1.35	2.15	1.76	2.64
Tooth extraction	93	2.13	1.79	2.38	4.39	3.58	5.39
Skin infection	91	1.60	1.25	1.85	3.02	2.46	3.72
Blood bilirubin increased	90	0.47	0.12	0.72	1.38	1.12	1.70
Rib fracture	89	0.80	0.45	1.05	1.74	1.41	2.14
Hypercalcaemia	89	0.66	0.31	0.92	1.58	1.29	1.95
Limb injury	89	0.46	0.11	0.71	1.37	1.12	1.69
Skin laceration	88	1.92	1.57	2.17	3.78	3.07	4.66
Myelodysplastic syndrome	87	0.85	0.50	1.11	1.81	1.46	2.23

Dementia	86	0.76	0.40	1.01	1.69	1.37	2.09
Mouth haemorrhage	85	2.25	1.89	2.51	4.75	3.84	5.89
Haematemesis	85	0.66	0.30	0.92	1.58	1.28	1.95
Hypophagia	85	0.40	0.04	0.66	1.32	1.07	1.63
Internal haemorrhage	84	1.02	0.66	1.28	2.02	1.63	2.51
Mental status changes	84	0.65	0.29	0.91	1.57	1.27	1.94
Gastric haemorrhage	83	1.84	1.47	2.10	3.57	2.88	4.43
Blood uric acid increased	82	2.51	2.15	2.78	5.71	4.59	7.10
Platelet count increased	82	1.12	0.75	1.38	2.17	1.75	2.70
Hair texture abnormal	82	0.94	0.58	1.20	1.92	1.55	2.39
Cytopenia	81	1.31	0.94	1.57	2.47	1.99	3.07
Hypersomnia	80	0.34	-0.03	0.61	1.27	1.02	1.58
Ventricular tachycardia	79	1.40	1.03	1.67	2.64	2.12	3.29
Cardiomyopathy	79	1.21	0.84	1.48	2.31	1.86	2.89
Kidney infection	79	0.83	0.46	1.10	1.78	1.42	2.21
Gastritis	79	0.39	0.02	0.66	1.31	1.05	1.64
Blood immunoglobulin M increased	78	5.40	5.03	5.67	42.26	33.40	53.47
Spinal operation	77	1.94	1.56	2.21	3.83	3.06	4.80
Malignant melanoma	77	1.18	0.81	1.46	2.27	1.82	2.84
Blood potassium increased	77	1.08	0.71	1.36	2.12	1.69	2.65
Eye operation	76	3.46	3.08	3.73	10.97	8.74	13.77
Chromaturia	75	0.80	0.42	1.08	1.75	1.39	2.19
Arthropod bite	74	2.17	1.79	2.45	4.51	3.59	5.67
Neoplasm	74	1.76	1.37	2.03	3.38	2.69	4.24
Pneumocystis jirovecii pneumonia	74	0.79	0.41	1.07	1.73	1.38	2.18
Upper limb fracture	74	0.68	0.30	0.96	1.60	1.28	2.02
Knee operation	73	2.38	1.99	2.65	5.19	4.12	6.54
Subarachnoid haemorrhage	73	1.60	1.21	1.88	3.04	2.41	3.82
Progressive multifocal leukoencephalopathy	73	1.47	1.08	1.75	2.76	2.20	3.48
Macular degeneration	73	1.10	0.71	1.38	2.14	1.70	2.69
Bacterial infection	73	0.58	0.20	0.86	1.50	1.19	1.89
Lacrimation increased	73	0.36	-0.03	0.63	1.28	1.02	1.61
Cardiac pacemaker insertion	71	2.94	2.54	3.22	7.66	6.06	9.69
Skin atrophy	71	2.17	1.78	2.45	4.50	3.56	5.69
Lymphoma	71	0.99	0.60	1.28	1.99	1.58	2.51
Haematocrit decreased	71	0.43	0.03	0.71	1.34	1.06	1.70
Pulmonary fibrosis	70	0.40	0.01	0.69	1.32	1.05	1.67
Haemolytic anaemia	69	1.76	1.36	2.05	3.38	2.67	4.29
Lymphoedema	69	1.45	1.05	1.74	2.74	2.16	3.47
Oral herpes	69	0.58	0.18	0.86	1.49	1.18	1.89
Respiratory tract congestion	69	0.52	0.12	0.81	1.43	1.13	1.82

Skin mass	68	1.83	1.43	2.12	3.56	2.81	4.52
Sudden death	68	1.76	1.35	2.05	3.38	2.66	4.29
Blood lactate dehydrogenase increased	66	0.71	0.30	1.00	1.64	1.29	2.08
Cardiac operation	65	2.45	2.03	2.74	5.45	4.27	6.96
Pneumothorax	65	0.80	0.39	1.10	1.74	1.37	2.22
Melaena	65	0.42	0.01	0.71	1.34	1.05	1.70
Prostatomegaly	64	2.55	2.14	2.85	5.86	4.58	7.50
Squamous cell carcinoma of skin	63	2.01	1.59	2.31	4.02	3.13	5.15
Pulmonary haemorrhage	62	1.66	1.24	1.96	3.16	2.46	4.06
Uveitis	62	0.99	0.57	1.29	1.99	1.55	2.55
Immune system disorder	62	0.98	0.56	1.28	1.97	1.53	2.53
Laceration	61	1.43	1.01	1.73	2.69	2.09	3.47
Lower limb fracture	61	0.70	0.27	1.00	1.62	1.26	2.09
Supraventricular tachycardia	59	1.49	1.06	1.80	2.81	2.18	3.63
Urinary bladder haemorrhage	58	3.55	3.11	3.86	11.69	9.01	15.17
Procedural haemorrhage	58	2.68	2.24	2.99	6.40	4.94	8.29
Cholecystectomy	58	1.64	1.20	1.95	3.11	2.40	4.03
Bacteraemia	57	0.74	0.30	1.06	1.67	1.29	2.17
Blood immunoglobulin M decreased	56	4.45	4.00	4.76	21.80	16.66	28.53
Ventricular fibrillation	56	1.37	0.93	1.69	2.59	1.99	3.37
Clostridium difficile colitis	56	0.62	0.18	0.94	1.54	1.18	2.00
Blood test abnormal	56	0.54	0.10	0.86	1.46	1.12	1.89
Pneumonia bacterial	55	1.38	0.93	1.70	2.60	1.99	3.39
Ejection fraction decreased	55	0.43	-0.02	0.75	1.35	1.03	1.76
Scratch	54	1.32	0.87	1.64	2.50	1.91	3.26
Diffuse large B-cell lymphoma	54	1.13	0.68	1.45	2.19	1.68	2.86
Subdural haemorrhage	53	2.74	2.29	3.07	6.69	5.10	8.78
Cardiac flutter	53	2.13	1.67	2.45	4.37	3.33	5.72
Product use complaint	53	1.72	1.26	2.04	3.29	2.51	4.31
Stent placement	53	1.61	1.15	1.94	3.05	2.33	4.00
Chronic graft versus host disease	52	2.06	1.60	2.39	4.17	3.17	5.48
Blindness unilateral	52	1.07	0.61	1.40	2.09	1.59	2.75
Coronary artery occlusion	52	1.02	0.56	1.35	2.03	1.55	2.67
Sinus congestion	52	0.54	0.07	0.87	1.45	1.10	1.90
Blood calcium increased	51	1.33	0.86	1.66	2.51	1.91	3.31
Scab	51	0.96	0.50	1.30	1.95	1.48	2.57
Graft versus host disease	51	0.96	0.50	1.29	1.95	1.48	2.56
Prostatic operation	50	4.69	4.22	5.02	25.79	19.36	34.35
Blood disorder	50	1.44	0.97	1.77	2.71	2.05	3.57

Eye infection	50	1.11	0.64	1.44	2.15	1.63	2.84
Nail growth abnormal	49	4.02	3.54	4.36	16.20	12.18	21.56
Scleroderma	49	2.33	1.86	2.67	5.03	3.80	6.67
Oral mucosal blistering	49	1.81	1.34	2.15	3.51	2.65	4.66
Hyperuricaemia	49	1.73	1.25	2.07	3.32	2.50	4.39
Pseudomonas infection	49	0.99	0.52	1.33	1.99	1.50	2.63
Nocturia	49	0.66	0.19	1.00	1.58	1.20	2.10
Blood immunoglobulin G decreased	48	3.15	2.67	3.50	8.89	6.68	11.83
Tooth fracture	48	0.85	0.37	1.20	1.81	1.36	2.40
Cerebral aspergillosis	47	3.46	2.98	3.81	11.01	8.24	14.70
Conjunctival haemorrhage	47	2.41	1.93	2.76	5.33	4.00	7.11
Spinal compression fracture	47	0.95	0.46	1.29	1.93	1.45	2.57
Cyst	47	0.66	0.18	1.01	1.59	1.19	2.11
Angina bullosa haemorrhagica	46	4.77	4.28	5.12	27.37	20.27	36.95
Limb operation	46	2.26	1.77	2.61	4.79	3.59	6.41
Immunosuppression	46	1.29	0.80	1.64	2.45	1.83	3.28
Wrist fracture	46	0.75	0.26	1.10	1.68	1.26	2.25
Malnutrition	45	0.82	0.32	1.17	1.76	1.31	2.36
Iron deficiency anaemia	45	0.54	0.04	0.89	1.45	1.08	1.94
Retinal haemorrhage	44	1.50	1.00	1.86	2.84	2.11	3.82
Therapy partial responder	44	0.77	0.27	1.13	1.70	1.27	2.29
Central nervous system lesion	44	0.45	-0.05	0.81	1.37	1.02	1.84
Splenic rupture	43	3.45	2.94	3.81	10.91	8.06	14.76
Oral surgery	43	3.22	2.71	3.58	9.31	6.88	12.59
Red blood cell count increased	43	2.36	1.85	2.72	5.14	3.80	6.94
Haemolysis	43	1.09	0.58	1.45	2.13	1.58	2.87
Peripheral coldness	43	0.48	-0.03	0.84	1.39	1.03	1.88
Vascular rupture	42	3.17	2.65	3.53	8.98	6.62	12.19
Ingrowing nail	42	2.72	2.20	3.08	6.58	4.85	8.92
Wound haemorrhage	42	2.09	1.57	2.45	4.24	3.13	5.75
Pneumonia fungal	42	2.05	1.53	2.41	4.13	3.05	5.60
Onychomycosis	42	1.44	0.93	1.81	2.72	2.01	3.68
Brain neoplasm	42	1.39	0.88	1.76	2.62	1.94	3.55
Haemorrhagic stroke	42	1.01	0.50	1.38	2.01	1.49	2.73
Aphthous ulcer	42	0.84	0.33	1.21	1.79	1.32	2.42
Oesophagitis	41	0.73	0.21	1.10	1.66	1.22	2.25
Facial paralysis	41	0.59	0.07	0.96	1.51	1.11	2.05
Dental operation	40	3.34	2.81	3.72	10.13	7.40	13.86
Colonoscopy	40	3.07	2.55	3.45	8.41	6.15	11.50
Lung infiltration	40	1.07	0.54	1.45	2.10	1.54	2.87
Ventricular extrasystoles	40	1.05	0.52	1.42	2.07	1.52	2.82
Bleeding time prolonged	39	3.71	3.17	4.09	13.05	9.49	17.95
Pericardial haemorrhage	39	2.68	2.14	3.06	6.40	4.66	8.78

Hip surgery	39	2.32	1.79	2.70	4.99	3.64	6.84
Aortic aneurysm	39	1.17	0.63	1.55	2.25	1.64	3.08
Haemarthrosis	39	0.72	0.19	1.10	1.65	1.20	2.26
Decreased activity	39	0.61	0.08	0.99	1.53	1.12	2.10
Prostatic specific antigen increased	39	0.54	0.00	0.92	1.45	1.06	1.98
Paralysis	39	0.47	-0.06	0.85	1.39	1.01	1.90
Benign prostatic hyperplasia	38	1.37	0.82	1.75	2.58	1.87	3.54
Pelvic fracture	38	1.18	0.64	1.57	2.27	1.65	3.12
Inguinal hernia	38	1.16	0.62	1.55	2.24	1.63	3.08
Myositis	38	1.07	0.53	1.46	2.11	1.53	2.90
Muscle strain	38	0.86	0.32	1.25	1.82	1.32	2.50
Blood magnesium decreased	38	0.66	0.12	1.05	1.58	1.15	2.18
Haemorrhage urinary tract	37	2.44	1.89	2.83	5.43	3.93	7.50
Brain abscess	37	2.19	1.64	2.58	4.56	3.30	6.31
Cardiac tamponade	37	1.92	1.37	2.31	3.78	2.74	5.23
Immune thrombocytopenic purpura	37	1.38	0.84	1.77	2.61	1.89	3.60
Aspiration	37	0.65	0.10	1.04	1.57	1.13	2.16
Hypertensive crisis	37	0.49	-0.06	0.88	1.40	1.02	1.94
Ulcer haemorrhage	36	1.28	0.73	1.68	2.44	1.76	3.38
Meningitis	36	1.17	0.62	1.57	2.25	1.63	3.13
Nerve compression	36	0.69	0.13	1.08	1.61	1.16	2.24
Ear discomfort	36	0.62	0.07	1.02	1.54	1.11	2.14
Mantle cell lymphoma recurrent	35	4.08	3.52	4.48	16.91	12.05	23.72
Red blood cell count abnormal	35	3.25	2.68	3.65	9.48	6.79	13.26
Gallbladder operation	35	2.70	2.13	3.10	6.49	4.65	9.05
Vitreous haemorrhage	35	2.48	1.91	2.88	5.57	3.99	7.77
Onychomadesis	35	1.75	1.18	2.15	3.36	2.41	4.68
Failure to thrive	35	1.67	1.11	2.07	3.19	2.29	4.45
Pleurisy	35	1.21	0.65	1.61	2.31	1.66	3.23
Faeces soft	35	0.95	0.38	1.35	1.93	1.38	2.69
Synovial cyst	35	0.82	0.26	1.22	1.76	1.27	2.46
Precancerous skin lesion	34	2.99	2.42	3.40	7.97	5.68	11.18
Sinus operation	34	2.70	2.13	3.11	6.49	4.63	9.11
Neutrophil count abnormal	34	2.27	1.70	2.68	4.83	3.44	6.77
Catheterisation cardiac	34	1.71	1.14	2.12	3.27	2.34	4.58
Heart rate abnormal	34	1.30	0.73	1.70	2.46	1.76	3.45
Haemorrhoidal haemorrhage	34	1.04	0.47	1.45	2.06	1.47	2.88
Gastrointestinal infection	34	0.68	0.11	1.09	1.60	1.14	2.24
Laryngitis	34	0.56	-0.02	0.96	1.47	1.05	2.06
Microsporidia infection	33	3.73	3.15	4.15	13.31	9.41	18.82
Malignant pleural effusion	33	2.82	2.24	3.23	7.06	5.01	9.96

Non-small cell lung cancer	33	2.10	1.52	2.51	4.28	3.04	6.03
Muscle haemorrhage	33	1.89	1.31	2.30	3.70	2.63	5.21
Cheilitis	33	1.81	1.23	2.22	3.50	2.48	4.93
Hyperkeratosis	33	1.19	0.61	1.60	2.28	1.62	3.21
Dizziness postural	33	0.59	0.01	1.00	1.51	1.07	2.12
Renal pain	33	0.57	-0.01	0.98	1.49	1.06	2.09
Chronic lymphocytic leukaemia recurrent	32	3.27	2.68	3.68	9.61	6.77	13.65
Lymph node pain	32	2.71	2.12	3.13	6.54	4.62	9.28
White blood cell disorder	32	2.18	1.59	2.60	4.53	3.20	6.41
Deafness unilateral	32	1.74	1.15	2.16	3.34	2.36	4.73
Fungal skin infection	32	1.60	1.01	2.02	3.03	2.14	4.29
Intestinal haemorrhage	32	1.25	0.66	1.66	2.37	1.67	3.35
Encephalitis	32	0.86	0.27	1.28	1.82	1.29	2.57
Post procedural infection	32	0.80	0.21	1.21	1.74	1.23	2.46
Rehabilitation therapy	31	2.22	1.62	2.65	4.66	3.27	6.64
Supraventricular extrasystoles	31	2.06	1.46	2.48	4.16	2.92	5.92
Protein total decreased	31	1.65	1.05	2.08	3.14	2.21	4.47
Furuncle	31	0.92	0.32	1.34	1.89	1.33	2.69
Rash pustular	31	0.81	0.21	1.24	1.75	1.23	2.50
Decubitus ulcer	31	0.64	0.04	1.06	1.56	1.09	2.21
Biopsy	30	3.77	3.16	4.20	13.61	9.46	19.59
Neck mass	30	1.58	0.97	2.01	2.98	2.08	4.27
Bacterial sepsis	30	1.37	0.76	1.80	2.59	1.81	3.71
Energy increased	30	1.14	0.53	1.57	2.20	1.54	3.15
Extrasystoles	30	1.13	0.52	1.57	2.19	1.53	3.14
Nasal dryness	30	1.05	0.44	1.48	2.07	1.44	2.96
Staphylococcal sepsis	30	0.98	0.37	1.41	1.97	1.38	2.82
Skin abrasion	30	0.92	0.31	1.35	1.89	1.32	2.71
Gingival pain	30	0.91	0.30	1.34	1.88	1.31	2.69
Immunoglobulins decreased	29	3.08	2.46	3.52	8.44	5.85	12.19
Haemoglobin increased	29	1.55	0.93	1.99	2.94	2.04	4.23
Lumbar vertebral fracture	29	1.53	0.91	1.97	2.90	2.01	4.17
Pulmonary alveolar haemorrhage	29	1.14	0.52	1.58	2.20	1.53	3.18
Micrographic skin surgery	28	5.14	4.51	5.59	35.24	23.45	52.95
Pneumonia cryptococcal	28	3.41	2.77	3.85	10.60	7.29	15.42
Cryptococcosis	28	2.36	1.73	2.81	5.14	3.54	7.46
Mucormycosis	28	1.16	0.53	1.61	2.24	1.54	3.25
Breast mass	28	1.03	0.40	1.47	2.04	1.41	2.95
Atrioventricular block	28	0.87	0.24	1.31	1.83	1.26	2.65
Hepatitis B	28	0.66	0.02	1.10	1.58	1.09	2.28
Bone marrow transplant	27	3.48	2.83	3.93	11.14	7.60	16.32
Dental care	27	2.85	2.21	3.31	7.23	4.95	10.58

Post procedural haematoma	27	2.42	1.78	2.88	5.36	3.67	7.84
Coronary arterial stent insertion	27	1.65	1.01	2.11	3.14	2.15	4.59
Coronavirus infection	27	1.03	0.39	1.49	2.05	1.40	2.99
Incorrect dosage administered	27	1.01	0.36	1.46	2.01	1.38	2.93
Nasal ulcer	26	2.54	1.89	3.01	5.83	3.96	8.59
Middle ear effusion	26	2.31	1.65	2.77	4.95	3.37	7.29
Cerebral haematoma	26	1.49	0.84	1.95	2.81	1.91	4.14
Guillain-Barre syndrome	26	1.43	0.77	1.89	2.69	1.83	3.96
Arterial occlusive disease	26	0.63	-0.02	1.09	1.55	1.05	2.28
Erysipelas	26	0.57	-0.09	1.03	1.48	1.01	2.18
Blood viscosity decreased	25	3.98	3.31	4.45	15.73	10.54	23.48
Meningitis cryptococcal	25	2.30	1.63	2.77	4.92	3.32	7.30
Renal mass	25	1.74	1.08	2.22	3.35	2.26	4.97
Ventricular arrhythmia	25	1.45	0.79	1.93	2.74	1.85	4.06
Cardiac valve disease	25	1.19	0.52	1.66	2.27	1.54	3.37
Hodgkin's disease	25	1.04	0.37	1.51	2.06	1.39	3.04
Congestive cardiomyopathy	25	0.97	0.30	1.44	1.96	1.32	2.90
COVID-19 pneumonia	25	0.66	-0.01	1.13	1.58	1.06	2.33
Dermatitis acneiform	25	0.63	-0.04	1.10	1.55	1.04	2.29
Haematotympanum	24	5.02	4.33	5.50	32.35	20.81	50.29
Arrhythmia supraventricular	24	3.32	2.63	3.80	9.96	6.64	14.93
Infective exacerbation of chronic obstructive airways disease	24	3.02	2.34	3.50	8.13	5.43	12.18
Haemorrhagic disorder	24	2.99	2.31	3.47	7.95	5.31	11.90
Traumatic haemorrhage	24	2.60	1.92	3.08	6.07	4.06	9.08
Chemotherapy	24	2.06	1.37	2.54	4.16	2.78	6.22
Poor peripheral circulation	24	1.20	0.52	1.68	2.30	1.54	3.43
SARS-CoV-2 test positive	24	0.97	0.29	1.45	1.96	1.31	2.93
Product blister packaging issue	23	3.35	2.65	3.84	10.21	6.75	15.44
Lymphocyte count abnormal	23	2.85	2.16	3.34	7.23	4.79	10.91
Bowen's disease	23	2.31	1.61	2.80	4.96	3.29	7.47
Nail discolouration	23	1.34	0.64	1.83	2.53	1.68	3.81
Compression fracture	23	1.15	0.45	1.64	2.21	1.47	3.33
Otitis media	23	1.11	0.41	1.60	2.15	1.43	3.24
Arthritis bacterial	23	1.03	0.33	1.52	2.04	1.35	3.07
Urine odour abnormal	23	0.87	0.17	1.36	1.83	1.21	2.75
Parosmia	23	0.76	0.06	1.25	1.70	1.13	2.55
Mucosal haemorrhage	22	2.72	2.00	3.22	6.57	4.31	10.00
Stem cell transplant	22	2.35	1.64	2.85	5.10	3.35	7.77
Nail infection	22	2.35	1.63	2.85	5.08	3.34	7.74
Post herpetic neuralgia	22	2.14	1.43	2.64	4.41	2.90	6.72

Pneumonia pseudomonal	22	2.06	1.35	2.56	4.18	2.75	6.36
Abdominal mass	22	1.66	0.95	2.16	3.17	2.08	4.82
Pyoderma gangrenosum	22	1.60	0.89	2.10	3.03	1.99	4.61
Non-cardiac chest pain	22	1.44	0.72	1.94	2.71	1.78	4.12
Atypical pneumonia	22	1.42	0.70	1.92	2.67	1.76	4.06
Aortic stenosis	22	1.37	0.66	1.87	2.59	1.70	3.94
Central venous catheterisation	22	1.26	0.54	1.76	2.39	1.57	3.63
Rash vesicular	22	1.19	0.48	1.69	2.29	1.50	3.48
Rhinovirus infection	22	0.88	0.16	1.38	1.84	1.21	2.79
Herpes virus infection	22	0.74	0.02	1.24	1.67	1.10	2.53
Iron deficiency	22	0.71	-0.01	1.21	1.63	1.07	2.48
Gastrointestinal motility disorder	22	0.65	-0.07	1.15	1.56	1.03	2.38
Traumatic haematoma	21	2.59	1.85	3.10	6.00	3.90	9.23
Multimorbidity	21	2.40	1.67	2.91	5.27	3.43	8.11
Platelet disorder	21	1.83	1.10	2.34	3.56	2.32	5.47
Eye contusion	21	1.79	1.06	2.30	3.46	2.25	5.32
Renal haemorrhage	21	1.75	1.02	2.27	3.37	2.19	5.18
Catheter site haemorrhage	21	1.43	0.70	1.94	2.69	1.75	4.14
Paranasal sinus hypersecretion	21	1.29	0.56	1.81	2.45	1.60	3.76
B-cell lymphoma	21	1.11	0.38	1.62	2.16	1.41	3.31
Renal function test abnormal	21	1.06	0.32	1.57	2.08	1.35	3.19
Urinary tract disorder	21	1.00	0.27	1.52	2.00	1.31	3.08
Melanocytic naevus	21	0.94	0.21	1.45	1.92	1.25	2.94
Humerus fracture	21	0.93	0.20	1.44	1.91	1.24	2.93
Facial bones fracture	21	0.90	0.17	1.41	1.87	1.22	2.86
Food poisoning	21	0.87	0.14	1.38	1.83	1.19	2.80
Graft versus host disease in skin	21	0.66	-0.07	1.17	1.58	1.03	2.42
Skin operation	20	4.37	3.62	4.89	20.67	13.10	32.62
Waldenstrom's macroglobulinaemia	20	3.66	2.91	4.18	12.62	8.08	19.71
Polymyositis	20	2.37	1.62	2.90	5.17	3.33	8.04
Pneumonia pneumococcal	20	2.08	1.33	2.60	4.22	2.72	6.56
Metabolic encephalopathy	20	1.40	0.65	1.93	2.64	1.70	4.10
Nonspecific reaction	20	1.22	0.47	1.74	2.33	1.50	3.61
Escherichia sepsis	20	1.14	0.39	1.66	2.20	1.42	3.41
Rosacea	20	0.86	0.11	1.38	1.81	1.17	2.81
Subcutaneous haematoma	19	2.28	1.51	2.82	4.85	3.09	7.62
Hyperphosphataemia	19	2.01	1.24	2.55	4.04	2.57	6.34
Ventricular dysfunction	19	2.00	1.23	2.54	4.01	2.56	6.30
Lip pain	19	1.86	1.09	2.39	3.62	2.31	5.69
Urinary tract obstruction	19	1.29	0.52	1.83	2.44	1.56	3.84
Macular oedema	19	0.84	0.07	1.38	1.79	1.14	2.81

Nail bed bleeding	18	3.22	2.43	3.77	9.30	5.83	14.84
Bladder operation	18	2.82	2.02	3.37	7.05	4.42	11.23
Animal bite	18	1.63	0.84	2.18	3.09	1.94	4.91
Renal neoplasm	18	1.57	0.78	2.12	2.97	1.87	4.72
Blood potassium abnormal	18	1.48	0.69	2.03	2.79	1.75	4.43
Tongue ulceration	18	1.14	0.34	1.69	2.20	1.38	3.49
Erythema nodosum	18	1.13	0.34	1.68	2.19	1.38	3.48
Coronary artery bypass	18	1.07	0.28	1.62	2.10	1.32	3.34
Aneurysm	18	0.89	0.10	1.44	1.86	1.17	2.95
Bundle branch block right	18	0.68	-0.11	1.23	1.60	1.01	2.55
Splenic haemorrhage	17	3.15	2.33	3.72	8.88	5.49	14.35
Infected bite	17	2.47	1.66	3.04	5.56	3.44	8.96
Central nervous system infection	17	2.33	1.51	2.89	5.02	3.11	8.09
Haematocrit abnormal	17	2.23	1.41	2.79	4.69	2.91	7.55
Spleen disorder	17	1.87	1.05	2.44	3.65	2.27	5.88
Sinus node dysfunction	17	1.53	0.71	2.09	2.88	1.79	4.64
Retroperitoneal haemorrhage	17	1.51	0.69	2.08	2.85	1.77	4.59
Prostatitis	17	1.24	0.42	1.80	2.36	1.46	3.79
Blood creatinine abnormal	17	1.12	0.31	1.69	2.18	1.35	3.51
Nocardiosis	17	0.93	0.11	1.49	1.90	1.18	3.06
Post procedural contusion	16	3.51	2.67	4.09	11.40	6.93	18.76
Hyphaema	16	3.37	2.53	3.95	10.34	6.29	16.99
Central nervous system haemorrhage	16	3.15	2.31	3.74	8.89	5.42	14.60
Noninfective gingivitis	16	2.09	1.25	2.68	4.26	2.61	6.97
Pulmonary mycosis	16	1.84	1.00	2.42	3.58	2.19	5.86
Intra-abdominal fluid collection	16	1.64	0.80	2.23	3.13	1.91	5.11
Intra-abdominal haemorrhage	16	1.43	0.59	2.01	2.69	1.65	4.40
Atrioventricular block second degree	16	1.41	0.57	1.99	2.66	1.63	4.35
Actinic keratosis	16	0.99	0.15	1.58	1.99	1.22	3.25
Tachyarrhythmia	16	0.90	0.06	1.49	1.87	1.14	3.05
Tongue discolouration	16	0.87	0.02	1.45	1.82	1.12	2.98
Faeces hard	16	0.82	-0.02	1.40	1.77	1.08	2.88
Mantle cell lymphoma refractory	15	4.22	3.35	4.82	18.59	10.95	31.57
Bladder neoplasm	15	2.16	1.29	2.76	4.47	2.69	7.43
Transplant	15	1.99	1.11	2.59	3.96	2.38	6.58
Oesophageal haemorrhage	15	1.84	0.97	2.44	3.58	2.15	5.95
Central nervous system lymphoma	15	1.81	0.94	2.41	3.51	2.11	5.83
Peritoneal haemorrhage	15	1.81	0.94	2.41	3.50	2.10	5.81

Splenic infarction	15	1.79	0.92	2.39	3.47	2.09	5.76
Lung neoplasm	15	1.43	0.56	2.03	2.69	1.62	4.47
Body temperature abnormal	15	1.37	0.50	1.97	2.58	1.56	4.29
Bladder pain	15	1.26	0.39	1.86	2.40	1.44	3.98
Lipoma	15	1.23	0.35	1.83	2.34	1.41	3.88
Shoulder operation	15	1.13	0.26	1.73	2.19	1.32	3.64
Lymphadenitis	15	1.03	0.16	1.63	2.04	1.23	3.38
Hepatosplenomegaly	15	0.96	0.09	1.56	1.94	1.17	3.23
Face injury	15	0.95	0.08	1.55	1.93	1.16	3.20
Skin fragility	15	0.76	-0.11	1.36	1.69	1.02	2.81
Blood immunoglobulin M abnormal	14	4.43	3.53	5.05	21.53	12.18	38.05
Haematospermia	14	3.03	2.12	3.65	8.15	4.80	13.84
Thyroid hormones decreased	14	2.35	1.45	2.97	5.10	3.01	8.64
Graft versus host disease in lung	14	2.30	1.40	2.92	4.93	2.91	8.35
Blood electrolytes abnormal	14	2.00	1.10	2.62	4.00	2.36	6.76
Abdominal lymphadenopathy	14	1.89	0.99	2.51	3.71	2.19	6.28
Cardiac ablation	14	1.88	0.98	2.50	3.68	2.17	6.22
Brain operation	14	1.87	0.96	2.49	3.65	2.16	6.17
Full blood count abnormal	14	1.56	0.66	2.18	2.95	1.74	4.99
Lip blister	14	1.48	0.57	2.10	2.78	1.65	4.71
Cystoid macular oedema	14	1.42	0.52	2.04	2.68	1.58	4.53
Empyema	14	1.32	0.42	1.94	2.50	1.48	4.23
Soft tissue infection	14	1.20	0.29	1.82	2.29	1.36	3.87
Carotid artery occlusion	14	1.18	0.28	1.81	2.27	1.34	3.84
Carotid artery stenosis	14	1.14	0.23	1.76	2.20	1.30	3.71
Limb mass	14	1.10	0.20	1.73	2.15	1.27	3.63
Tumour haemorrhage	14	1.08	0.18	1.70	2.11	1.25	3.57
Appendicectomy	14	1.07	0.16	1.69	2.10	1.24	3.54
Aplasia pure red cell	14	1.06	0.16	1.68	2.08	1.23	3.52
Muscle rupture	14	0.80	-0.10	1.42	1.74	1.03	2.95
Chronic lymphocytic leukaemia transformation	13	3.81	2.88	4.46	14.07	8.05	24.61
Ear operation	13	3.65	2.71	4.29	12.53	7.19	21.84
Biopsy lung	13	3.31	2.37	3.95	9.90	5.70	17.19
Immunodeficiency common variable	13	3.24	2.31	3.89	9.48	5.46	16.44
Lymphoma transformation	13	3.15	2.21	3.79	8.87	5.12	15.38
Aspiration pleural cavity	13	2.51	1.57	3.15	5.68	3.28	9.81
Nail operation	13	2.48	1.54	3.12	5.58	3.23	9.65
Intensive care	13	2.41	1.48	3.06	5.33	3.09	9.21
Abdominal neoplasm	13	2.40	1.46	3.04	5.26	3.04	9.09
Post procedural swelling	13	2.16	1.22	2.81	4.48	2.59	7.73

Animal scratch	13	2.05	1.11	2.70	4.15	2.40	7.16
Discharge	13	2.04	1.10	2.68	4.11	2.38	7.10
Pharyngeal haemorrhage	13	1.99	1.05	2.63	3.97	2.30	6.85
Protein total abnormal	13	1.73	0.79	2.37	3.31	1.92	5.72
Eyelid margin crusting	13	1.70	0.76	2.34	3.24	1.88	5.60
Pulmonary sepsis	13	1.58	0.64	2.23	3.00	1.74	5.17
Urine flow decreased	13	1.53	0.60	2.18	2.90	1.68	5.00
Tympanic membrane perforation	13	1.52	0.58	2.16	2.87	1.66	4.95
Acute febrile neutrophilic dermatosis	13	1.44	0.50	2.09	2.72	1.58	4.69
Emergency care	13	1.33	0.39	1.97	2.52	1.46	4.34
Acute sinusitis	13	1.13	0.19	1.77	2.19	1.27	3.78
Adenocarcinoma	13	1.06	0.12	1.70	2.09	1.21	3.60
Metastatic malignant melanoma	13	1.02	0.08	1.66	2.02	1.17	3.49
Hepatitis E	13	1.01	0.07	1.65	2.01	1.17	3.46
Chapped lips	13	0.82	-0.12	1.47	1.77	1.03	3.05
Escherichia bacteraemia	13	0.82	-0.12	1.46	1.76	1.02	3.03
Transurethral prostatectomy	12	3.25	2.27	3.92	9.53	5.37	16.91
Nasal operation	12	3.09	2.11	3.76	8.50	4.80	15.07
Nail ridging	12	2.82	1.84	3.49	7.07	3.99	12.52
Lung operation	12	2.65	1.67	3.32	6.27	3.55	11.09
Central venous catheter removal	12	2.34	1.36	3.01	5.06	2.87	8.94
Axillary mass	12	2.20	1.22	2.87	4.59	2.60	8.11
Pneumococcal sepsis	12	2.11	1.13	2.78	4.32	2.45	7.63
Dacryostenosis acquired	12	2.08	1.10	2.74	4.22	2.39	7.44
Intestinal mass	12	1.80	0.82	2.47	3.48	1.97	6.14
Pneumonia streptococcal	12	1.65	0.67	2.32	3.14	1.78	5.53
Infectious pleural effusion	12	1.47	0.50	2.14	2.78	1.58	4.90
Infected skin ulcer	12	1.24	0.26	1.91	2.37	1.34	4.17
Lung cancer metastatic	12	1.22	0.24	1.88	2.32	1.32	4.10
Endodontic procedure	12	1.17	0.19	1.83	2.25	1.27	3.96
Otorrhoea	12	1.16	0.19	1.83	2.24	1.27	3.95
Near death experience	12	1.12	0.15	1.79	2.18	1.24	3.84
Eye injury	12	1.03	0.05	1.70	2.04	1.16	3.60
Oesophageal stenosis	12	1.03	0.05	1.69	2.04	1.15	3.59
Pulse abnormal	12	1.01	0.03	1.68	2.02	1.14	3.56
Iridocyclitis	12	1.00	0.02	1.67	2.00	1.14	3.53
Iritis	12	0.95	-0.03	1.62	1.93	1.10	3.41
Facial operation	11	3.90	2.87	4.59	14.92	8.06	27.61
Oral blood blister	11	3.74	2.72	4.44	13.40	7.29	24.63
Splenic haematoma	11	3.13	2.11	3.82	8.75	4.81	15.93

Blood viscosity increased	11	2.97	1.94	3.66	7.82	4.30	14.21
Capillary fragility	11	2.76	1.74	3.46	6.79	3.74	12.33
Toe operation	11	2.39	1.37	3.09	5.25	2.90	9.52
Penile haemorrhage	11	2.38	1.35	3.07	5.20	2.87	9.42
Vaccination complication	11	2.10	1.07	2.79	4.27	2.36	7.74
Testicular swelling	11	2.07	1.04	2.76	4.19	2.31	7.59
Therapeutic procedure	11	1.94	0.91	2.63	3.82	2.11	6.92
Enterovirus infection	11	1.87	0.84	2.56	3.64	2.01	6.60
Dental implantation	11	1.81	0.79	2.50	3.51	1.94	6.35
Product difficult to swallow	11	1.71	0.69	2.41	3.28	1.81	5.94
Listeriosis	11	1.69	0.67	2.38	3.23	1.78	5.84
Intracranial mass	11	1.57	0.54	2.26	2.96	1.64	5.36
Bladder prolapse	11	1.56	0.54	2.26	2.96	1.63	5.35
Hyperleukocytosis	11	1.29	0.26	1.98	2.44	1.35	4.41
Neoplasm skin	11	1.25	0.23	1.95	2.38	1.32	4.31
Sinusitis fungal	11	1.22	0.20	1.91	2.33	1.29	4.21
Pancreatic cyst	11	1.20	0.18	1.90	2.30	1.27	4.16
Aortic valve stenosis	11	0.86	-0.17	1.55	1.81	1.00	3.28
Leukostasis syndrome	10	3.52	2.45	4.25	11.49	6.09	21.68
Chronic lymphocytic leukaemia refractory	10	3.43	2.35	4.16	10.79	5.73	20.32
Lumbar puncture	10	3.19	2.12	3.92	9.14	4.87	17.16
Prostatectomy	10	3.12	2.05	3.85	8.71	4.65	16.33
Platelet dysfunction	10	2.98	1.91	3.71	7.91	4.23	14.81
Dermatitis infected	10	2.76	1.69	3.49	6.80	3.64	12.71
Blood immunoglobulin A decreased	10	2.56	1.49	3.29	5.91	3.16	11.03
Discouragement	10	2.29	1.22	3.02	4.90	2.63	9.14
Spontaneous haemorrhage	10	2.27	1.19	3.00	4.82	2.59	8.99
Splenectomy	10	2.11	1.03	2.83	4.31	2.31	8.03
Red blood cell abnormality	10	2.10	1.02	2.83	4.29	2.30	7.99
Neuroendocrine carcinoma of the skin	10	2.09	1.02	2.82	4.27	2.29	7.95
Colon neoplasm	10	2.06	0.98	2.78	4.16	2.23	7.75
Gastrointestinal polyp haemorrhage	10	1.95	0.87	2.67	3.86	2.07	7.19
Prostate cancer recurrent	10	1.93	0.85	2.65	3.80	2.04	7.09
Gastrointestinal ulcer haemorrhage	10	1.72	0.65	2.45	3.31	1.77	6.16
Chondrocalcinosis pyrophosphate	10	1.71	0.63	2.43	3.26	1.75	6.07
Peptic ulcer haemorrhage	10	1.68	0.60	2.41	3.21	1.72	5.98
Coagulation time prolonged	10	1.66	0.59	2.39	3.17	1.70	5.90
Cystitis noninfective	10	1.60	0.53	2.33	3.04	1.63	5.66

Oral mucosal erythema	10	1.58	0.51	2.31	3.00	1.61	5.59
Gastrointestinal tube insertion	10	1.55	0.47	2.27	2.92	1.57	5.43
Calculus bladder	10	1.49	0.42	2.22	2.82	1.51	5.24
Mechanical ventilation	10	1.49	0.41	2.21	2.80	1.50	5.21
Graft versus host disease in liver	10	1.40	0.32	2.13	2.64	1.42	4.91
Rotator cuff repair	10	1.36	0.29	2.09	2.58	1.38	4.80
Wound infection staphylococcal	10	1.28	0.20	2.00	2.42	1.30	4.51
Tongue blistering	10	1.22	0.14	1.94	2.33	1.25	4.33
Pneumonia staphylococcal	10	1.21	0.13	1.94	2.31	1.24	4.31
Chronic graft versus host disease in skin	10	1.21	0.13	1.94	2.31	1.24	4.30
Mitral valve disease	10	1.14	0.06	1.86	2.20	1.18	4.09
Diabetic complication	10	1.03	-0.05	1.75	2.04	1.09	3.79
Purpura senile	9	3.46	2.32	4.22	10.97	5.62	21.42
Lymph node palpable	9	3.30	2.17	4.07	9.88	5.08	19.23
Scleral haemorrhage	9	3.25	2.11	4.01	9.52	4.90	18.52
Hyperviscosity syndrome	9	3.21	2.07	3.97	9.24	4.76	17.95
Cerebral fungal infection	9	3.18	2.04	3.94	9.04	4.65	17.56
Allergy to arthropod bite	9	3.13	1.99	3.89	8.75	4.51	16.99
Beta 2 microglobulin increased	9	2.97	1.83	3.73	7.84	4.05	15.20
Bladder mass	9	2.81	1.67	3.57	7.00	3.62	13.54
Lichenification	9	2.72	1.58	3.48	6.57	3.40	12.71
Tumour flare	9	2.45	1.31	3.21	5.46	2.83	10.55
Transfusion reaction	9	2.13	0.99	2.89	4.37	2.27	8.43
Cellulitis staphylococcal	9	2.11	0.97	2.87	4.30	2.23	8.30
Chylothorax	9	2.01	0.87	2.77	4.03	2.09	7.77
Retroperitoneal lymphadenopathy	9	1.85	0.71	2.61	3.61	1.87	6.95
Stomach mass	9	1.78	0.64	2.54	3.44	1.78	6.62
Demyelinating polyneuropathy	9	1.73	0.59	2.49	3.32	1.72	6.40
Squamous cell carcinoma of lung	9	1.72	0.58	2.48	3.28	1.70	6.33
Disseminated cryptococcosis	9	1.66	0.52	2.42	3.15	1.64	6.07
Campylobacter gastroenteritis	9	1.64	0.50	2.40	3.12	1.62	6.01
Cardiac fibrillation	9	1.63	0.49	2.39	3.09	1.61	5.96
Fungal sepsis	9	1.59	0.45	2.36	3.02	1.57	5.81
Aortic valve disease	9	1.52	0.39	2.29	2.88	1.49	5.54
Staphylococcal skin infection	9	1.46	0.33	2.23	2.76	1.43	5.32
Uterine polyp	9	1.42	0.28	2.18	2.68	1.39	5.16
Intra-abdominal haematoma	9	1.41	0.27	2.17	2.65	1.38	5.10

Gingival recession	9	1.38	0.24	2.14	2.60	1.35	5.00
Glycosylated haemoglobin decreased	9	1.37	0.23	2.13	2.58	1.34	4.96
Otitis externa	9	1.36	0.22	2.12	2.56	1.33	4.93
Peripheral artery occlusion	9	1.11	-0.03	1.87	2.16	1.12	4.16
Vitreous detachment	9	1.03	-0.11	1.79	2.04	1.06	3.93
Hernia repair	9	0.97	-0.17	1.73	1.96	1.02	3.77
Postmenopausal haemorrhage	9	0.95	-0.19	1.71	1.93	1.00	3.72
Pneumocystis jirovecii infection	9	0.95	-0.19	1.71	1.93	1.00	3.71
Lymphocyte adoptive therapy	8	3.79	2.57	4.59	13.79	6.56	29.01
Capillary permeability increased	8	3.71	2.50	4.51	13.10	6.30	27.23
Eyelid operation	8	3.27	2.06	4.07	9.64	4.75	19.56
Ventricular tachyarrhythmia	8	2.68	1.47	3.48	6.40	3.18	12.89
Prostatic haemorrhage	8	2.63	1.42	3.44	6.21	3.09	12.49
Biopsy bone marrow	8	2.56	1.35	3.36	5.90	2.94	11.87
Skin graft	8	2.45	1.24	3.25	5.46	2.71	10.96
Lymph gland infection	8	2.32	1.11	3.13	5.00	2.49	10.05
Gastric operation	8	2.29	1.08	3.10	4.90	2.44	9.84
Oncologic complication	8	2.09	0.88	2.89	4.25	2.12	8.54
Spontaneous haematoma	8	2.05	0.84	2.85	4.15	2.07	8.32
Serratia infection	8	2.01	0.80	2.82	4.04	2.01	8.11
Retinal vascular occlusion	8	1.96	0.75	2.76	3.89	1.94	7.81
Colon operation	8	1.95	0.74	2.75	3.87	1.93	7.75
Oesophageal rupture	8	1.92	0.71	2.72	3.78	1.89	7.59
Blister rupture	8	1.91	0.70	2.71	3.76	1.88	7.54
Aortic valve replacement	8	1.91	0.69	2.71	3.75	1.87	7.51
Product packaging difficult to open	8	1.83	0.62	2.63	3.55	1.77	7.12
Prostate infection	8	1.82	0.61	2.63	3.54	1.77	7.10
Sarcoma	8	1.82	0.61	2.62	3.53	1.76	7.09
Arteriovenous malformation	8	1.72	0.50	2.52	3.29	1.64	6.59
West Nile viral infection	8	1.57	0.36	2.38	2.98	1.49	5.97
Medical device implantation	8	1.45	0.24	2.26	2.74	1.37	5.49
Blood electrolytes decreased	8	1.41	0.20	2.22	2.66	1.33	5.33
Streptococcal sepsis	8	1.41	0.20	2.21	2.65	1.32	5.32
Blood bilirubin abnormal	8	1.34	0.13	2.15	2.54	1.27	5.08
Joint warmth	8	1.31	0.09	2.11	2.48	1.24	4.96
Obstruction gastric	8	1.29	0.08	2.09	2.45	1.22	4.90
Streptococcal bacteraemia	8	1.26	0.04	2.06	2.39	1.19	4.79
Systemic infection	8	1.17	-0.04	1.98	2.25	1.13	4.52
Early satiety	8	1.17	-0.04	1.97	2.25	1.12	4.50
Oral mucosal eruption	8	1.13	-0.08	1.93	2.19	1.09	4.38

Small cell lung cancer	8	1.13	-0.08	1.93	2.19	1.09	4.38
Axillary pain	8	1.12	-0.09	1.92	2.17	1.08	4.35
Cerebellar haemorrhage	8	1.08	-0.13	1.88	2.12	1.06	4.24
Biopsy prostate	7	3.41	2.11	4.26	10.62	4.94	22.86
Ureteric compression	7	3.41	2.10	4.26	10.60	4.93	22.79
Blood viscosity abnormal	7	3.13	1.83	3.98	8.76	4.12	18.63
Immunoglobulins abnormal	7	3.12	1.82	3.97	8.68	4.08	18.46
Biopsy skin	7	2.96	1.66	3.81	7.79	3.67	16.51
Ear tube insertion	7	2.82	1.52	3.67	7.07	3.34	14.97
Ear neoplasm	7	2.67	1.37	3.53	6.38	3.02	13.49
Human rhinovirus test positive	7	2.60	1.30	3.45	6.06	2.87	12.80
Breast haemorrhage	7	2.57	1.27	3.42	5.95	2.82	12.56
Oral contusion	7	2.41	1.11	3.26	5.31	2.52	11.19
Thyroid operation	7	2.36	1.06	3.21	5.14	2.44	10.84
Pyogenic granuloma	7	2.34	1.03	3.19	5.05	2.39	10.64
Soft tissue haemorrhage	7	2.34	1.03	3.19	5.05	2.39	10.64
Autoimmune neutropenia	7	2.13	0.83	2.98	4.38	2.08	9.22
Endoscopy	7	2.08	0.78	2.94	4.24	2.01	8.93
Pharyngeal mass	7	2.07	0.77	2.93	4.21	2.00	8.87
Cystitis escherichia	7	2.02	0.72	2.87	4.06	1.93	8.55
Thyroxine increased	7	2.00	0.70	2.86	4.01	1.90	8.44
Palliative care	7	1.85	0.55	2.70	3.61	1.72	7.60
Gouty arthritis	7	1.79	0.49	2.64	3.46	1.64	7.27
Bladder catheterisation	7	1.74	0.44	2.60	3.35	1.59	7.05
Chronic myelomonocytic leukaemia	7	1.73	0.43	2.58	3.32	1.58	6.99
Blood sodium abnormal	7	1.68	0.38	2.53	3.21	1.52	6.74
Lower respiratory tract infection fungal	7	1.57	0.26	2.42	2.96	1.41	6.23
Haematoma muscle	7	1.45	0.14	2.30	2.73	1.30	5.73
Neovascular age-related macular degeneration	7	1.42	0.11	2.27	2.67	1.27	5.61
Carotid artery disease	7	1.26	-0.04	2.11	2.40	1.14	5.04
Radiotherapy	7	1.24	-0.06	2.09	2.37	1.13	4.97
Hepatic infection	7	1.24	-0.06	2.09	2.36	1.12	4.96
Gastric pH decreased	7	1.22	-0.08	2.07	2.33	1.11	4.89
Streptococcus test positive	7	1.17	-0.13	2.02	2.25	1.07	4.73
Lip injury	7	1.15	-0.15	2.00	2.22	1.06	4.67
Platelet transfusion	7	1.13	-0.17	1.98	2.19	1.04	4.59
Wrist surgery	7	1.12	-0.19	1.97	2.17	1.03	4.55
Mallory-Weiss syndrome	7	1.08	-0.22	1.93	2.11	1.01	4.44
Scrotal haematocoele	6	3.12	1.70	4.03	8.69	3.83	19.72
Prostatic mass	6	3.04	1.63	3.95	8.23	3.64	18.62

Pharyngeal operation	6	2.89	1.47	3.80	7.39	3.28	16.66
Marginal zone lymphoma	6	2.87	1.46	3.78	7.31	3.25	16.47
Cold type haemolytic anaemia	6	2.84	1.43	3.75	7.17	3.19	16.15
Cystitis klebsiella	6	2.77	1.36	3.69	6.84	3.04	15.39
Rectal neoplasm	6	2.77	1.35	3.68	6.82	3.03	15.33
B-cell small lymphocytic lymphoma	6	2.69	1.27	3.60	6.44	2.87	14.47
Atypical mycobacterial pneumonia	6	2.66	1.24	3.57	6.30	2.81	14.14
Infusion	6	2.49	1.08	3.41	5.63	2.51	12.63
Pleural infection	6	2.47	1.05	3.38	5.53	2.47	12.40
Catheter site cellulitis	6	2.46	1.04	3.37	5.50	2.45	12.32
Post procedural haematuria	6	2.42	1.01	3.33	5.36	2.39	12.01
Renal cyst haemorrhage	6	2.39	0.98	3.31	5.26	2.35	11.78
Artificial crown procedure	6	2.30	0.89	3.21	4.93	2.20	11.04
Vessel puncture site bruise	6	2.25	0.83	3.16	4.75	2.12	10.64
Spinal cord haemorrhage	6	2.22	0.80	3.13	4.66	2.08	10.42
Serositis	6	2.14	0.73	3.06	4.42	1.98	9.89
Urinary tract infection staphylococcal	6	2.05	0.64	2.96	4.15	1.85	9.27
Thrombotic stroke	6	2.04	0.62	2.95	4.11	1.84	9.19
Allergy to vaccine	6	1.97	0.55	2.88	3.91	1.75	8.74
Listeria sepsis	6	1.92	0.51	2.84	3.80	1.70	8.48
Abdominal cavity drainage	6	1.89	0.47	2.80	3.70	1.66	8.28
Cellulitis orbital	6	1.89	0.47	2.80	3.70	1.66	8.28
Suture insertion	6	1.82	0.41	2.73	3.53	1.58	7.89
Throat lesion	6	1.77	0.35	2.68	3.40	1.52	7.60
Head and neck cancer	6	1.76	0.35	2.68	3.40	1.52	7.59
Skin neoplasm excision	6	1.75	0.34	2.66	3.37	1.51	7.52
IIIrd nerve paralysis	6	1.69	0.27	2.60	3.22	1.44	7.19
Critical illness	6	1.68	0.26	2.59	3.20	1.43	7.15
Renal haematoma	6	1.67	0.26	2.58	3.19	1.43	7.11
Blister infected	6	1.65	0.23	2.56	3.13	1.40	6.99
Ingrown hair	6	1.61	0.19	2.52	3.05	1.37	6.81
Bone marrow infiltration	6	1.59	0.18	2.50	3.02	1.35	6.73
Bite	6	1.51	0.10	2.43	2.86	1.28	6.38
Pneumatosis	6	1.51	0.10	2.42	2.85	1.28	6.36
Implantable defibrillator insertion	6	1.49	0.07	2.40	2.81	1.26	6.26
Mastoiditis	6	1.46	0.05	2.37	2.76	1.24	6.15
Hepatic haemorrhage	6	1.46	0.05	2.37	2.76	1.23	6.15
Polypectomy	6	1.42	0.01	2.33	2.68	1.20	5.98
Excessive cerumen production	6	1.39	-0.03	2.30	2.62	1.17	5.84
Amputation	6	1.30	-0.12	2.21	2.46	1.10	5.48

Burkitt's lymphoma	6	1.25	-0.17	2.16	2.38	1.07	5.30
Skin texture abnormal	6	1.21	-0.21	2.12	2.31	1.03	5.15
Heart valve replacement	6	1.17	-0.25	2.08	2.25	1.01	5.01
Thyroid hormones increased	6	1.17	-0.25	2.08	2.25	1.01	5.01
Bing-Neel syndrome	5	3.38	1.82	4.36	10.40	3.56	30.44
Iodine uptake abnormal	5	3.34	1.78	4.32	10.11	3.75	27.24
Nail cuticle fissure	5	3.12	1.56	4.10	8.68	3.50	21.56
Pseudohyperkalaemia	5	3.07	1.51	4.06	8.40	3.40	20.77
Fungal abscess central nervous system	5	3.02	1.46	4.00	8.10	3.29	19.93
Stomatitis haemorrhagic	5	2.92	1.36	3.90	7.57	3.09	18.53
Venous operation	5	2.72	1.16	3.70	6.58	2.71	16.02
Meningitis enteroviral	5	2.57	1.01	3.55	5.93	2.44	14.40
Abnormal clotting factor	5	2.50	0.94	3.48	5.65	2.33	13.71
Breast haematoma	5	2.45	0.89	3.44	5.47	2.26	13.26
Haemorrhagic ascites	5	2.37	0.80	3.35	5.16	2.13	12.49
Pleuropericarditis	5	2.37	0.80	3.35	5.16	2.13	12.49
Penile ulceration	5	2.27	0.71	3.26	4.83	2.00	11.68
Blood uric acid abnormal	5	2.23	0.67	3.22	4.70	1.94	11.36
Cardiac pacemaker replacement	5	2.18	0.62	3.17	4.53	1.88	10.96
Pneumonitis chemical	5	2.16	0.59	3.14	4.46	1.84	10.77
Hypertensive urgency	5	2.12	0.56	3.11	4.36	1.80	10.52
Haemobilia	5	2.07	0.50	3.05	4.19	1.73	10.11
Brain cancer metastatic	5	2.05	0.48	3.03	4.13	1.71	9.98
Nail bed disorder	5	1.98	0.42	2.96	3.95	1.63	9.52
Breast operation	5	1.90	0.34	2.88	3.73	1.54	8.99
Ocular neoplasm	5	1.84	0.28	2.82	3.58	1.48	8.63
Post thrombotic syndrome	5	1.80	0.24	2.79	3.49	1.45	8.41
Renal surgery	5	1.80	0.24	2.78	3.48	1.44	8.39
Graft versus host disease in eye	5	1.75	0.19	2.73	3.36	1.39	8.11
Metastatic squamous cell carcinoma	5	1.71	0.15	2.70	3.28	1.36	7.90
Bone abscess	5	1.70	0.14	2.69	3.25	1.35	7.84
Paraneoplastic syndrome	5	1.68	0.12	2.67	3.21	1.33	7.74
Oral mucosal discolouration	5	1.66	0.10	2.65	3.17	1.31	7.64
Groin infection	5	1.66	0.10	2.65	3.17	1.31	7.63
Periorbital cellulitis	5	1.64	0.08	2.63	3.12	1.29	7.52
Positron emission tomogram abnormal	5	1.62	0.06	2.61	3.07	1.28	7.41
Post procedural sepsis	5	1.56	0.00	2.55	2.96	1.23	7.12
Scrotal oedema	5	1.56	-0.01	2.54	2.94	1.22	7.09
Haematoma infection	5	1.55	-0.01	2.54	2.93	1.22	7.06

Transient global amnesia	5	1.54	-0.02	2.53	2.91	1.21	7.01
Lobar pneumonia	5	1.51	-0.05	2.50	2.86	1.19	6.88
Cauda equina syndrome	5	1.51	-0.05	2.50	2.85	1.18	6.87
Oesophagitis ulcerative	5	1.49	-0.07	2.48	2.82	1.17	6.79
Soft tissue mass	5	1.44	-0.12	2.43	2.72	1.13	6.55
Occult blood	5	1.44	-0.13	2.42	2.71	1.12	6.52
Urinary tract infection pseudomonal	5	1.42	-0.14	2.41	2.68	1.11	6.45
Alcohol intolerance	5	1.39	-0.17	2.38	2.63	1.09	6.33
Embolic cerebral infarction	5	1.36	-0.20	2.34	2.56	1.06	6.18
Bronchial carcinoma	5	1.35	-0.22	2.33	2.54	1.06	6.13
Gingival erythema	5	1.31	-0.25	2.30	2.48	1.03	5.98
Spinal cord neoplasm	5	1.28	-0.28	2.27	2.43	1.01	5.86
Prostatic urethral lift procedure	4	3.05	1.28	4.13	8.27	2.81	24.32
Globulin abnormal	4	2.85	1.08	3.93	7.19	2.62	19.76
Spleen atrophy	4	2.83	1.07	3.91	7.13	2.60	19.56
Testicular haemorrhage	4	2.78	1.01	3.86	6.86	2.51	18.73
Cochlea implant	4	2.73	0.96	3.81	6.63	2.43	18.04
Human anaplasmosis	4	2.72	0.95	3.80	6.59	2.42	17.93
Stent removal	4	2.69	0.93	3.77	6.46	2.38	17.56
Ocular retrobulbar haemorrhage	4	2.62	0.86	3.70	6.16	2.27	16.68
Clostridium difficile sepsis	4	2.62	0.85	3.70	6.14	2.27	16.64
Biopsy lymph gland	4	2.51	0.75	3.59	5.71	2.12	15.42
Eyelid bleeding	4	2.51	0.74	3.59	5.68	2.11	15.34
Renal artery stent placement	4	2.50	0.73	3.58	5.66	2.10	15.27
Bone marrow necrosis	4	2.47	0.70	3.55	5.54	2.05	14.93
Paraneoplastic pemphigus	4	2.45	0.68	3.52	5.45	2.02	14.69
Platelet function test abnormal	4	2.35	0.58	3.43	5.10	1.89	13.71
Retroperitoneal mass	4	2.29	0.53	3.37	4.90	1.82	13.19
Glaucoma surgery	4	2.29	0.52	3.36	4.87	1.81	13.10
Mole excision	4	2.26	0.49	3.33	4.78	1.78	12.83
Cryptococcal fungaemia	4	2.21	0.44	3.29	4.62	1.72	12.40
Periorbital haemorrhage	4	2.20	0.44	3.28	4.60	1.71	12.35
Nail bed infection	4	2.17	0.40	3.25	4.49	1.67	12.06
Scrotal infection	4	2.17	0.40	3.24	4.49	1.67	12.04
Upper respiratory tract infection bacterial	4	2.12	0.35	3.20	4.34	1.62	11.66
Biopsy bone marrow abnormal	4	2.11	0.34	3.19	4.31	1.60	11.55
Cavernous sinus thrombosis	4	2.10	0.34	3.18	4.30	1.60	11.53
Lip squamous cell carcinoma	4	2.09	0.33	3.17	4.26	1.59	11.42
Spinal cord haematoma	4	2.07	0.31	3.15	4.21	1.57	11.28

Tongue haematoma	4	2.06	0.29	3.14	4.16	1.55	11.16
Fluid replacement	4	2.05	0.29	3.13	4.15	1.55	11.14
Eye infection viral	4	2.01	0.24	3.09	4.03	1.50	10.79
Polyserositis	4	1.97	0.20	3.05	3.91	1.46	10.49
Oesophageal food impaction	4	1.97	0.20	3.04	3.91	1.46	10.47
Ureteral stent insertion	4	1.90	0.14	2.98	3.73	1.39	10.00
Synovial rupture	4	1.87	0.11	2.95	3.66	1.37	9.81
Pulmonary necrosis	4	1.85	0.09	2.93	3.61	1.35	9.68
Skin ulcer haemorrhage	4	1.84	0.08	2.92	3.59	1.34	9.60
Medial tibial stress syndrome	4	1.82	0.05	2.90	3.53	1.32	9.44
Vertebroplasty	4	1.82	0.05	2.90	3.53	1.32	9.44
Tongue eruption	4	1.75	-0.01	2.83	3.37	1.26	9.02
Cataract operation complication	4	1.73	-0.03	2.81	3.32	1.24	8.89
Catheter site haematoma	4	1.72	-0.04	2.80	3.30	1.23	8.83
Blood creatine abnormal	4	1.69	-0.08	2.77	3.22	1.20	8.62
Aspergilloma	4	1.68	-0.08	2.76	3.21	1.20	8.59
Brain neoplasm benign	4	1.67	-0.10	2.75	3.18	1.19	8.51
Enterobacter bacteraemia	4	1.65	-0.11	2.73	3.15	1.18	8.42
Medical induction of coma	4	1.65	-0.12	2.73	3.14	1.17	8.39
Gastroduodenal ulcer	4	1.64	-0.12	2.72	3.12	1.17	8.35
Postoperative thrombosis	4	1.60	-0.17	2.67	3.02	1.13	8.08
Aspergillus test positive	4	1.59	-0.18	2.66	3.00	1.12	8.03
Neutrophilic dermatosis	4	1.53	-0.24	2.61	2.89	1.08	7.72
Tumour rupture	4	1.51	-0.26	2.59	2.85	1.06	7.61
Bronchopneumopathy	4	1.51	-0.26	2.59	2.84	1.06	7.60
Bladder obstruction	4	1.50	-0.27	2.58	2.82	1.06	7.55
Bladder irritation	4	1.50	-0.27	2.57	2.82	1.05	7.54
Central nervous system fungal infection	4	1.47	-0.29	2.55	2.78	1.04	7.42
Mammogram abnormal	4	1.44	-0.33	2.52	2.71	1.01	7.23
Post procedural inflammation	4	1.43	-0.33	2.51	2.70	1.01	7.21
Pneumonia haemophilus	4	1.42	-0.34	2.50	2.68	1.00	7.18
